# Base pair probability estimates improve the prediction accuracy of RNA non-canonical base pairs

**DOI:** 10.1371/journal.pcbi.1005827

**Published:** 2017-11-06

**Authors:** Michael F. Sloma, David H. Mathews

**Affiliations:** 1 Department of Biochemistry & Biophysics and Center for RNA Biology, University of Rochester Medical Center, Rochester, NY, United States of America; 2 Department of Biostatistics & Computational Biology, University of Rochester Medical Center, Rochester, NY, United States of America; University of Missouri, UNITED STATES

## Abstract

Prediction of RNA tertiary structure from sequence is an important problem, but generating accurate structure models for even short sequences remains difficult. Predictions of RNA tertiary structure tend to be least accurate in loop regions, where non-canonical pairs are important for determining the details of structure. Non-canonical pairs can be predicted using a knowledge-based model of structure that scores nucleotide cyclic motifs, or NCMs. In this work, a partition function algorithm is introduced that allows the estimation of base pairing probabilities for both canonical and non-canonical interactions. Pairs that are predicted to be probable are more likely to be found in the true structure than pairs of lower probability. Pair probability estimates can be further improved by predicting the structure conserved across multiple homologous sequences using the TurboFold algorithm. These pairing probabilities, used in concert with prior knowledge of the canonical secondary structure, allow accurate inference of non-canonical pairs, an important step towards accurate prediction of the full tertiary structure. Software to predict non-canonical base pairs and pairing probabilities is now provided as part of the RNAstructure software package.

## Author summary

Three dimensional RNA structure prediction methods are not yet able to accurately model the base pairs that are not in standard A-form helices, called non-canonical pairs. Non-canonical base pairs are crucial in determining the conformations of structures, but available algorithms to identify them have limited accuracy. We developed a new method, CycleFold, that can identify non-canonical base pairs using statistical methods that have proven successful in predicting A-form helices. Additionally, CycleFold incorporates evolutionary conservation to further improve accuracy. CycleFold provides a dramatic improvement in accuracy over previously available methods, and its output could be used to refine three dimensional structure predictions from any modeling software.

## Introduction

### RNA tertiary structure prediction

RNA plays a central role in all of life, acting as both a carrier of genetic information and as an active participant in numerous cellular processes, including pre-mRNA splicing and gene regulation via modulation of transcription and translation [1]. Determining the structure of an RNA is crucial to understand its function, and provides opportunities to design drugs that target the molecule, but the structure of the vast majority of RNA molecules is entirely unknown. As of 2016, the Protein Databank contained coordinates for 1233 RNA structures and 5914 protein-nucleic acid complexes [2], covering less than 2000 structural classes [3]. At the same time, the ENCODE project identified over 9000 long non-coding RNAs in the human genome alone [4]. Experimental methods to determine RNA tertiary structures at atomic resolution, such as NMR, X-ray crystallography, and cryo-electron microscopy, remain expensive, difficult, and time-consuming, so computational structure prediction methods, which have proven effective in predicting unknown protein structures [5,6], are desirable to help investigators understand RNAs of unknown structure.

Given sufficient human expertise, manual structure modeling can produce high-quality predictions of structure. For example, the structure of the Group I self-splicing intron was modeled and subsequently many of the atomic interactions were confirmed by crystal structures [7–10]. More recently, a wide range of computational methods were developed to automatically predict RNA structure. These methods include fragment assembly [11–14], all-atom modeling with constraints from sequence comparison and experimental information using molecular mechanics [15,16], coarse-grained molecular simulation [17–22], coarse-grained helix-as-a-stick models [23–25], and homology modeling using a known structure [26]. All of these methods can use sparse information from experimental methods or low-resolution computational structure prediction, such as prediction of secondary structure, to reduce the search space of the tertiary structure problem and improve accuracy.

In general, automated structure prediction methods can identify the global fold of an RNA, but modeling of loop regions is frequently inaccurate [27]. These poorly-predicted regions are often highly ordered, containing specific arrangements of non-canonical base pairs [27,28]. In this work, a method was developed for accurate inference of non-canonical base pairs in loops, which could provide additional restraints for modeling the tertiary structure. Non-canonical pair predictions could also aid in the identification of RNA modules, which are self-contained RNA motifs with known structure [29].

### Prediction of non-canonical base pairs

The canonical RNA secondary structure (the set of A-U, G-C, and G-U wobble pairs in an RNA structure) can be predicted using a free energy change model developed from optical melting experiments on short RNA sequences [30] combined with a dynamic programming algorithm that efficiently searches the space of possible structures to identify the best structure according to the model [31]. This method implicitly considers the free energy change of some non-canonical pairs, such as mismatched nucleotides at the end of helices, but it does not explicitly make predictions of non-canonical pairs.

Non-canonical base pairs can be explicitly predicted using an alternative model of RNA structure that scores Nucleotide Cyclic Motifs, or NCMs [12,32], instead of thermodynamic nearest neighbor parameters. An NCM is a cycle in a graph that represents the structure of an RNA, where the vertices represent nucleotides and the edges represent either covalent backbone interactions or base-pairing. In these graphs, both canonical and non-canonical base pairs are treated as edges, and a nucleotide is restricted to pair with at most one other nucleotide. The score of an NCM is determined using a training set of tertiary structures from the Protein Data Bank [2,12]. The score relates to the frequency with which a particular sequence is observed in an NCM of a particular size and by the frequency with which types of NCMs appear next to one another, and the scores are cast as free energy changes. Prediction using this model is implemented in the MC-Fold program [12]. Two subsequent implementations of this calculation, MC-Fold-DP [33] and MC-Flashfold [34], use a dynamic programming algorithm, making structure calculations feasible for long sequences (>150 nucleotides). Although NCM-based methods offer the advantage of predicting an extended secondary structure that includes non-canonical base pairs, they have the significant drawback in that they are that are less accurate at predicting the canonical secondary structure than prior thermodynamics-based methods, especially for sequences longer than about 50 nucleotides [33]. Therefore, methods to increase the accuracy of predicted structures, or to determine what parts of a predicted structure are more likely to be correct, are desirable.

### Partition functions

Both MC-Fold and MC-Fold-DP search for the best structure for a sequence given the scoring model, or for a set of suboptimal structures within a certain scoring increment of the best structure. An alternative approach to structure prediction has proven highly useful in the prediction of canonical secondary structure: predicting base pairing probabilities using an RNA partition function [35]. Instead of producing the best structure, or a set of likely structures, the partition function is used to calculate the probability of base pairing between each pair of nucleotides in the sequences. Additionally, the probability that a given nucleotide is base paired can be calculated from this information. Here it is shown, with benchmarks of structure prediction accuracy with sequences with known structure, that pairs (canonical and non-canonical) predicted to have high probability are more likely to be present in the true structure [36]. This method allows assessment of confidence in a predicted secondary structure [36], estimates of accessibility of a region of secondary structure [37–39], prediction of pseudoknots [40,41], and identification of single-nucleotide polymorphisms that are expected to affect structure [42–45].

### Conserved structure prediction

Computational methods to identify conserved base pairs have also proven useful in prediction of canonical pairs in RNA because structure is conserved to greater extent than sequence [46]. Pairs that are predicted to be conserved in multiple homologs are more likely to be correctly predicted. One method for predicting conserved structures is the TurboFold algorithm [47], which takes unaligned homologous sequences, with unknown structure, as input and uses a probabilistic Hidden Markov Model alignment and the structure partition function to estimate the pairing probability for conserved pairs.

### Summary

This work demonstrates that methods that improve the prediction accuracy of canonical secondary structure also improve the prediction accuracy of non-canonical pairs, i.e. the extended secondary structure, using the NCM model. In this work, an RNA partition function algorithm was implemented for the NCM model of structure, and benchmarks were performed that show pairs predicted to be highly probable are more likely to be accurate predictions. Additionally, the TurboFold algorithm [47] was implemented for use with the NCM-based pair probability predictions to identify non-canonical pairs conserved in a set of homologous sequences, further improving the accuracy of extended secondary structure prediction.

## Results

### A partition function for the NCM structure model

**Fig 1:**
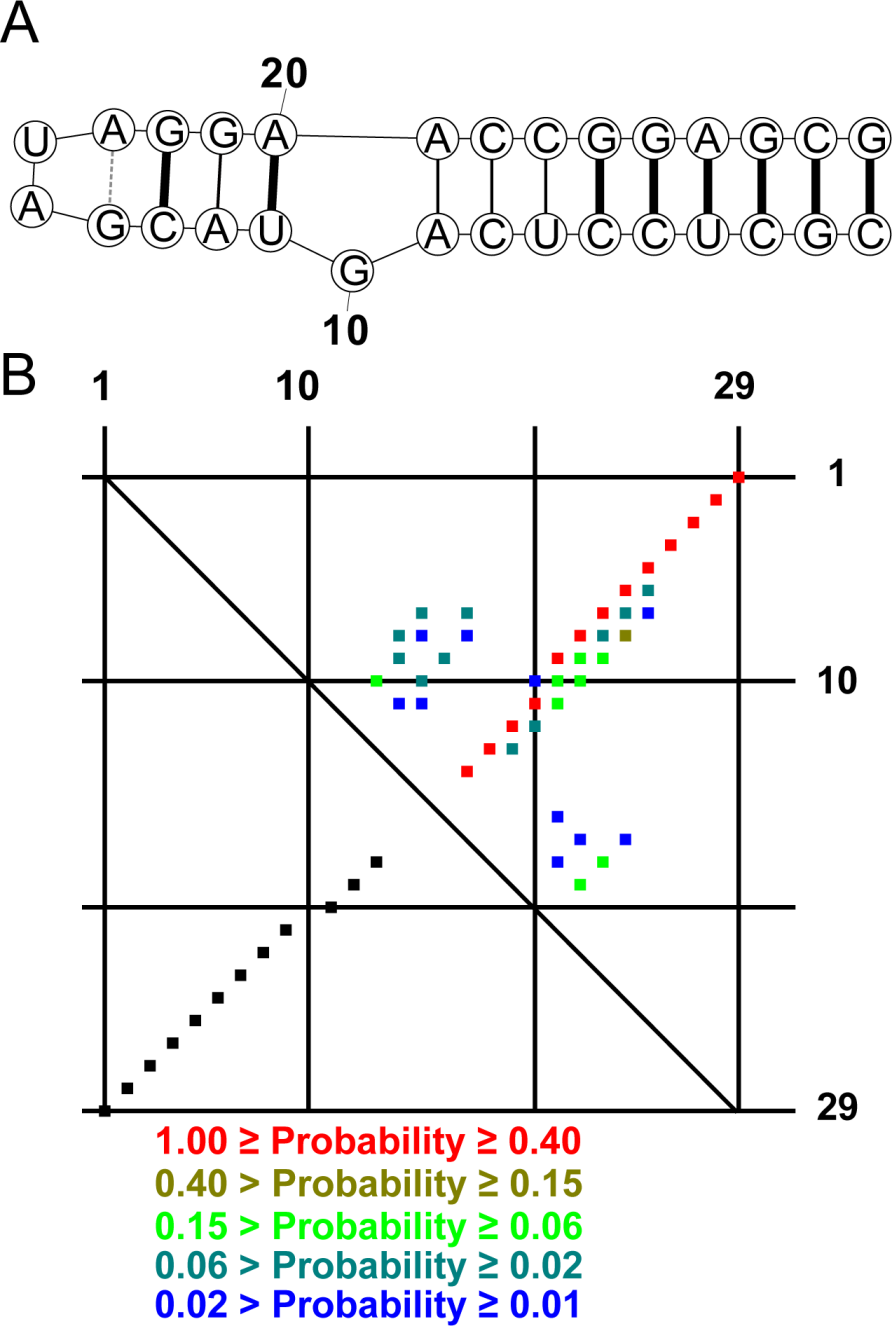
Prediction of extended secondary structure with CycleFold. (A) A predicted structure for a Sarcin-Ricin loop sequence form *Rattus norvegicus* [50] using CycleFold with the MFE algorithm. Correctly predicted canonical pairs are drawn with heavy black lines, correctly predicted non-canonical pairs are light black lines, and the incorrectly predicted non-canonical pair is shown with a gray dashed line. The G-A pair at the base of the tetraloop is not present in the reference structure because the 3’ A is not stacked on the subsequent G, but is instead in contact with a protein, Restrictocin. (B) The probability dot plot calculated using Cyclefold with the partition function algorithm. The upper right triangle shows pairs with estimated probabilities > 0.01, color-coded by pairing probability. The lower left triangle shows the pairs that are present in the reference structure. Each dot represents a single base pair, and nucleotide index (starting with 1 at the 5’ end) is shown along the x and y axes.

A partition function for the NCM-based model was implemented in a new software tool, CycleFold. This program takes an RNA sequence as input and produces either a minimum free energy structure (MFE; analogously to MC-Fold and MC-Fold-DP) or a matrix of pairing probabilities between each nucleotide in the sequence, considering the possibility of non-canonical base pairing (Fig 1). The maximum expected accuracy (MEA) method [48,49] or the ProbKnot method [40] can be used to generate structures from tables of pair probabilities. MEA structures are assembled from pairs of higher probability than those in MFE structures, and can be more accurate than MFE structures. ProbKnot assembles structures that can contain pseudoknotted base pairs and are composed of base pairs of nucleotides with mutually maximal pairing probabilities. These are pairs of nucleotides where, for each nucleotide, the probability of pairing to its partner is higher than the probability of pairing to any other nucleotide.

The formulation of the partition function algorithm is similar to the minimum free energy algorithm for the NCM model [33], with the restriction that care must be taken to count each structure exactly once. The algorithm is implemented using dynamic programming, and it has the same time and memory complexity as the partition function for canonical base pairs (O(N^3^) and O(N^2^), respectively, where N is the sequence length), with larger constant factors because of the greater variety of possible pairs that must be considered. A detailed description of the algorithm is provided in Materials and Methods, below. Because the dynamic programming search is equivalent for both the partition function and the calculation of the minimum energy structure, the source code for CycleFold implements only one generic set of dynamic programming recursions that are used by both algorithms.

### Benchmarks of structure prediction

To test the accuracy of CycleFold compared to previously available methods, a test set of structures from the RNA STRAND database [51] was assembled. Structures were chosen that were derived from coordinates in the Protein Data Bank [2], and that were not used as part of the MC-Fold training data [12]. In all, 154 structures met these criteria (S1 Table). Structures for each sequence were predicted with CycleFold, MC-Fold MC-Fold-DP, and the Fold program from the RNAstructure software package, which predicts canonical base pairs only using the conventional nearest neighbor rules [30], and the predictions were compared to the reference structure (S2 Table). Performance was measured using sensitivity, which is the fraction of known base pairs that were correctly predicted, and positive predictive value (PPV), which is the fraction of predicted pairs that were correct.

### MFE prediction accuracy for canonical base pairs

**Fig 2:**
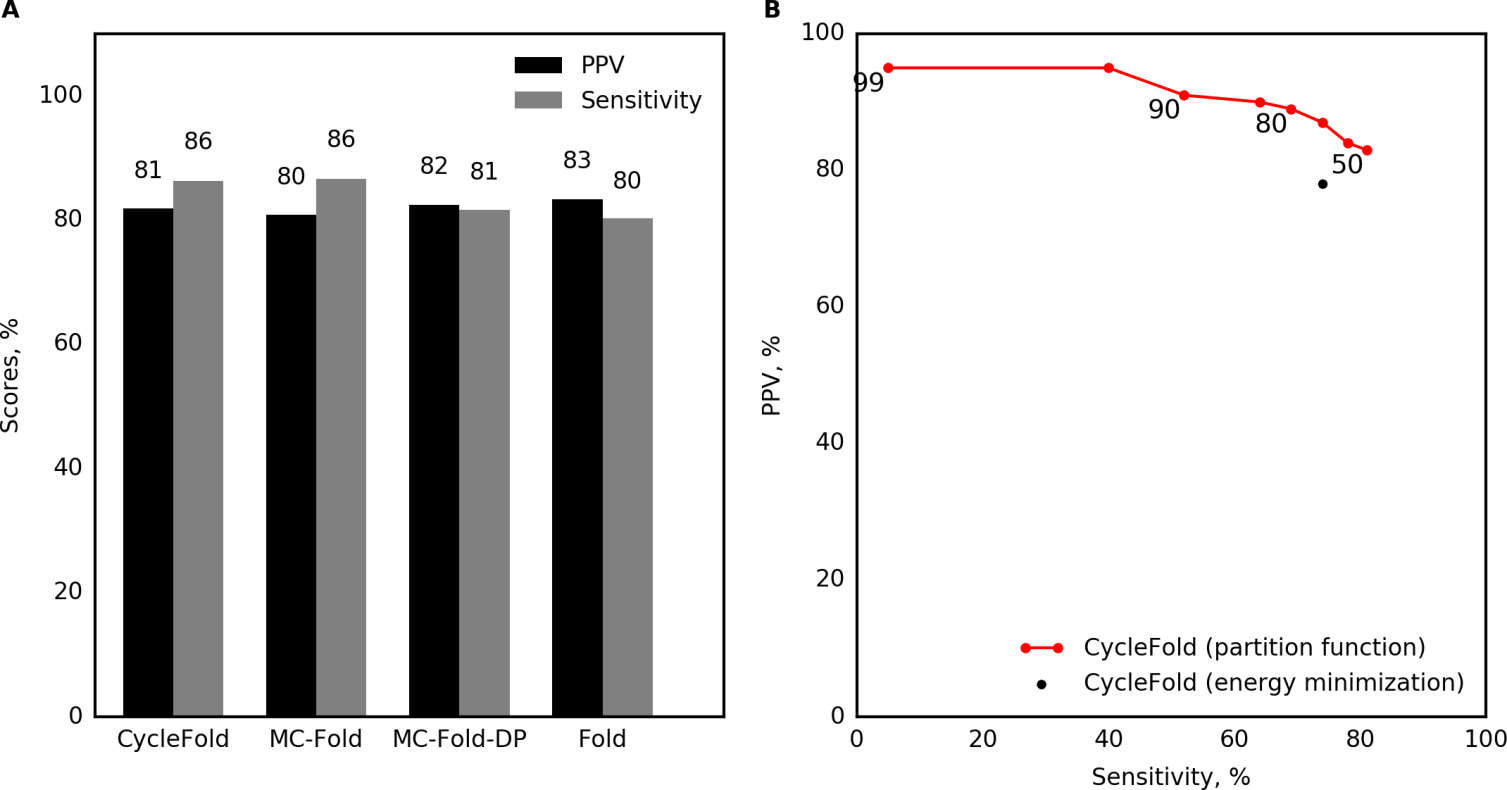
Benchmark of single-sequence prediction of canonical base pairs. (A) Prediction accuracy of the lowest free energy structure, evaluated on canonical pairs. (B) Prediction with CycleFold, using structures composed of highly probable canonical pairs. Sensitivity and PPV are reported for structures with probability greater than a threshold labeled on the plot). This demonstrates that the threshold stringency provides a tradeoff in terms of sensitivity and PPV.

The first benchmarks examined the prediction of canonical base pairs, defined as the set of A-U, G-C, and G-U pairs with at least three nucleotides between them. As expected, the three programs that use the NCM model have similar performance, although there are small differences in performance because of details of the implementation of each program (Fig 2A). Some of these differences are statistically significant with the type I error rate of 0.05. CycleFold and MC-Fold have significantly higher sensitivity compared to MC-Fold-DP, although the difference in performance is small. CycleFold and MC-Fold, but not MC-Fold-DP, have a small but statistically significant decrease in PPV compared to prediction with Fold (p < 0.05; S3 Table)

### Improvement of canonical pair predictions using the NCM partition function

Using the NCM partition function implemented in CycleFold, structures were assembled of canonical pairs that had probabilities higher than a threshold [36]. Structures composed of highly probable pairs had higher positive predictive value than structures predicted with the MFE method. For example, structures composed only of pairs with estimated pairing probability of 0.95 or higher had an average PPV of 95.4%, while still retaining an average sensitivity of 40.1% (Fig 2B; S2 Table), and all probability thresholds higher than 0.5 resulted in a significantly increased PPV over CycleFold-MFE (p<0.05; S4 Table).

### Accuracy of non-canonical pair prediction

**Fig 3:**
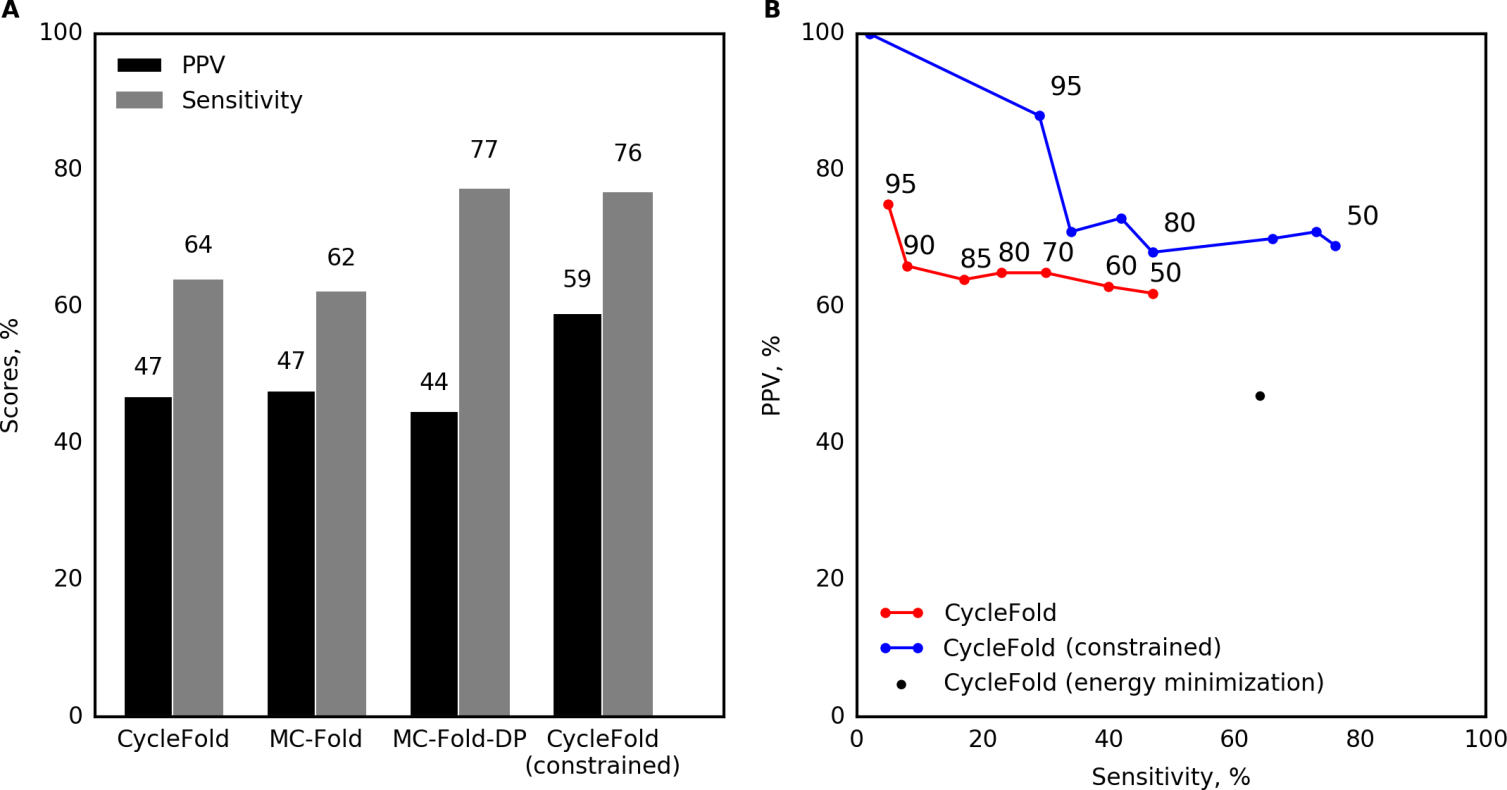
Benchmark of single-sequence prediction of non-canonical base pairs. (A) Prediction accuracy of the lowest free energy structure, evaluated on non-canonical pairs. This includes a calculation where CycleFold is constrained to include the known canonical base pairs to illustrate the performance of the NCM approach when canonical base pairs are known. (B) Prediction with CycleFold, using structures composed of highly probable non-canonical pairs. Sensitivity and PPV are reported for structures with probability greater than a specified threshold (labeled on the plot). This demonstrates that the threshold stringency provides a tradeoff in terms of sensitivity and PPV.

Next, prediction accuracy for non-canonical pairs (pairs other than the canonical A-U, G-C, or G-U pairs, or that did not have at least three nucleotides in between) was measured. Prediction of non-canonical pairs (Fig 3A) had poorer accuracy than for canonical pairs. CycleFold scored 47.0% for PPV, meaning that less than half of the predicted non-canonical pairs were correct, and 64.0% for sensitivity. CycleFold performed similarly to MC-Fold and MC-Fold-DP (Fig 3A). As with the canonical base pairs, CycleFold and MC-Fold have significantly higher sensitivity compared to MC-Fold-DP (p<0.05).

Non-canonical pairs are less frequent in the database of sequences with known structure than canonical base pairs. Therefore, using the NCM approach, they contribute less stability to the folding of a sequence to a structure that contains them than do the canonical pairs. The pair probabilities for non-canonical pairs, however, can still be high, i.e. close to 1, because pair probabilities reflect the competition of base pairs with alternative pairs with the same nucleotide. Sets of adjacent pairs can be difficult to compete with alternative structures, especially if some of the pairs are canonical pairs.

Assembling structures composed only of pairs above a specific pairing probability threshold identified non-canonical pairs with improved positive predictive value (Fig 3B). For example, structures composed of pairs with probability greater than 0.9 had PPV of 66.7%, albeit with a sensitivity of only 8.7%. This improvement in PPV is not statistically significant for a benchmark of this size (p > 0.05; S6 Table).

### Improvement of structure prediction using canonical structure constraints

Historically, the location of the canonical base pairs has been known with high accuracy before the tertiary structure was solved, so the canonical secondary structure can be used to help infer tertiary structure, including non-canonical base pairs [7,52]. CycleFold (as well as MC-Fold and MC-Fold-DP) is able to accept a set of input pairs, and predict structures that are required to contain those pairs. This reduces the search space of the problem, and therefore is expected to improve the accuracy of prediction. Using CycleFold, the structures in the test dataset were predicted using constraints that force the canonical base pairs to occur. This improved the prediction of non-canonical pairs with the MFE method to a PPV of 59.2% and a sensitivity of 76.8%, sufficiently accurate to develop hypotheses about structure (Fig 3A; S2 Table). This represents a significant improvement in PPV as compared to unconstrained MFE prediction using CycleFold (p < 0.05; S5 Table).

The CycleFold partition function calculation can also be constrained to require specified base pairs. Assembling structures with highly probable pairs using constraints in the partition function calculation improved the accuracy even further, with a PPV of 88.9% and a sensitivity of 29.7% for pairs with probability of 0.95 or greater. Pairing probability thresholds of 0.5 to 0.7 provide a significant improvement in PPV over MFE prediction. At higher thresholds, too few pairs are predicted, and there is insufficient statistical power to for a significant result (Fig 3B; S7 Table).

### Identification of conserved non-canonical pairs using the TurboFold algorithm

**Fig 4:**
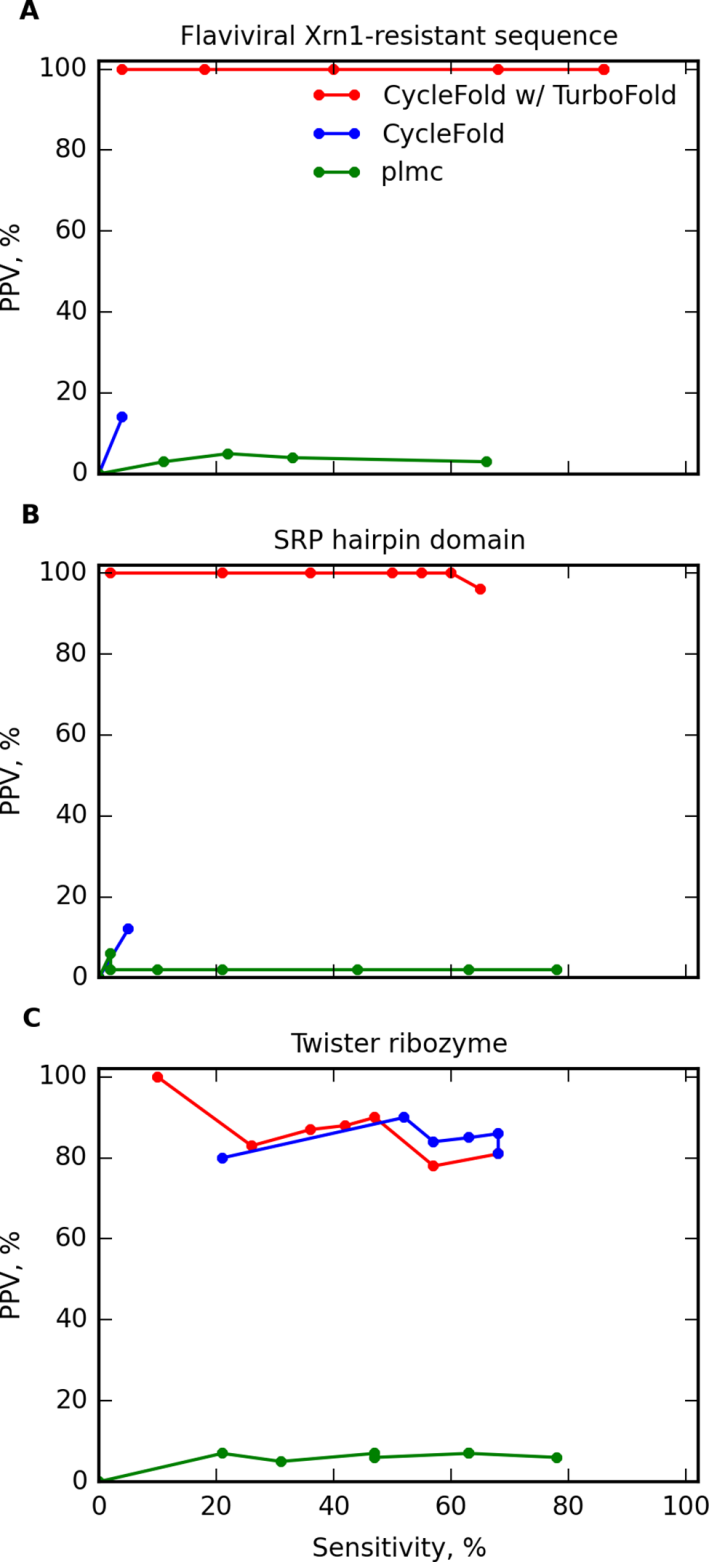
Prediction of canonical base pairs by predicting a conserved structure using multiple homologous sequences for (A) an MVE virus nuclease resistant RNA [53], (B) a *D*. *radiodurans* SRP hairpin domain [54], and (C) a *O*. *sativa* Twister ribozyme [55]. Prediction accuracy is shown for structures composed of highly probable pairs using information from a single sequence (blue) or a TurboFold calculation with 10 sequences (red). Also shown is prediction accuracy using evolutionary couplings from the plmc program [16] (green).

**Fig 5:**
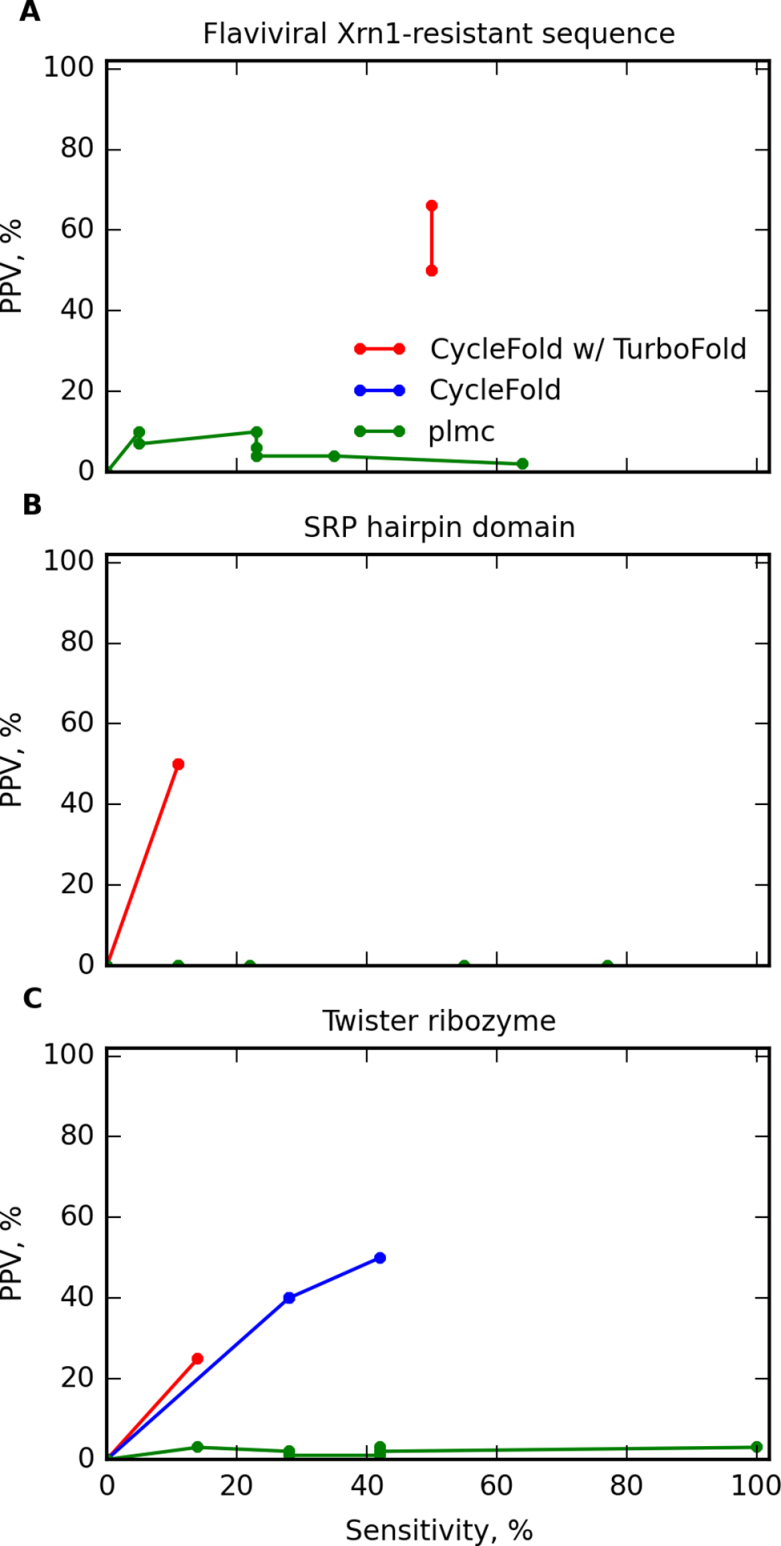
Prediction of non-canonical base pairs by predicting the conserved structure with multiple homologous sequences for (A) an MVE virus nuclease resistant RNA, (B) a *D*. *radiodurans* SRP hairpin domain, and (C) a *O*. *sativa* Twister ribozyme. Prediction accuracy is shown for structures composed of highly probable pairs using information from a single sequence (blue) or a TurboFold calculation on 10 sequences (red). In panels A and B, no blue line is present because the single sequence prediction did not correctly predict any pairs. Also shown is prediction using evolutionary couplings from the plmc program [16] (green).

Another way to improve the prediction of RNA structure is to predict the conserved structure using multiple homologous sequences. The NCM partition function was used to implement the TurboFold algorithm [47] as an additional mode in CycleFold. This algorithm uses pair probabilities for multiple sequences, in addition to a predicted, probabilistic alignment between each pair of sequences, to iteratively improve the estimated base pair probabilities for each sequence (see Materials and Methods). Three recent RNA crystal structures were assembled to test whether TurboFold improved the quality of predicted structures: a nuclease-resistant sequence from a Murray Valley Encephalitis virus 3’ UTR [53], a *Deinococcus radiodurans* signal recognition particle hairpin domain [54], and the structure of the Twister ribozyme from *Oryza sativa* [55]. The native structures as shown in the crystal structures are provided as Supplementary S1 Fig. Structures were chosen that were not present in the training data for the NCM model parameters, did not have large inserted folding domains that were removed from the crystallization construct, and were not riboswitches. Nine homologous sequences were chosen at random for each structure from a set of known homologs, for a total of 10 input sequences, and consensus pair probabilities were predicted using CycleFold’s TurboFold mode. The consensus pair probabilities were compared to the pair probabilities calculated only from the crystallized sequence using the NCM partition function for canonical pairs (Fig 4A–4C) and non-canonical pairs (Fig 5A–5C). In comparison, the plmc program, which identifies nucleotides that are associated using evolutionary couplings [16], was benchmarked, using the full set of aligned homologs as input.

In these benchmarks for predicting conserved pairs, base triples are considered two separate pairs, where one pair can be a canonical pair. The plmc program is able to predict triple interactions as a set of two pairs [16], although CycleFold is not able to predict base triples. Therefore, the plmc can achieve higher sensitivity on the benchmark.

For canonical pairs, in two cases, the nuclease-resistant RNA and the SRP RNA, the structure was predicted poorly using the single sequence, with under 10% sensitivity and PPV for the single-sequence predictions. In these cases, TurboFold was able to dramatically improve the prediction accuracy, with sensitivity over 60% and PPV over 90%. In the remaining case, the Twister ribozyme, the structure was reasonably accurate using both the single sequence and the TurboFold method. TurboFold offered a slight increase in PPV, with a slight decrease in sensitivity. In comparison of CycleFold to plmc, it was not possible to conclude that either program had better sensitivity, but CycleFold offered a significantly better PPV (p<0.05; S8 Table).

The prediction of non-canonical pairs had lower accuracy, but followed the same trend (Fig 5). The prediction using a single sequence did not correctly predict any non-canonical pairs for the nuclease-resistant RNA or the SRP RNA. Prediction with TurboFold increased the accuracy to a PPV of over 50% for each. In the case of the Twister ribozyme, TurboFold did not increase the accuracy over the single sequence prediction. As with the canonical base pairs, CycleFold had lower sensitivity but higher PPV than plmc. Due to the small number of sequences that could be used in this benchmark, however, there is insufficient statistical power to conclude that these differences are statistically significant using a type I error rate of 0.05 (S8 Table).

## Discussion

This work shows that conserved non-canonical base pairs can be identified in a set of unaligned homologous sequences. It is not surprising that this is the case; it has been long appreciated that RNA structure is more conserved than sequence. Furthermore, tertiary structure models had been developed in the past, most notably for the self-splicing group I intron, by manual identification of conserved interactions [7]. This work is notable as an automated method that builds on prior work on secondary structure prediction.

Prior work had emphasized the relative inaccuracy of the prediction of the NCM model when used with a dynamic programming algorithm to search the folding space [33]. Here, it is shown that the additional information from a partition function calculation can be used to identify the predicted pairs more likely to be correct. Additionally, constraining the structure prediction with knowledge of the canonical base pairs, along with a partition function calculation to find the most likely pairs, results in highly accurate predictions of non-canonical pairs. Finally, conserved non-canonical pairs can be predicted using the TurboFold algorithm.

An important limitation of the algorithm used in CycleFold is that it ignores the possibility of pseudoknotted pairs, that is, configurations of base pairs that are non-nested. However, it would be possible to predict pseudoknotted pairs using methods like ProbKnot, which has been provided as an optional mode for CycleFold. Additionally, prior work showed that the TurboFold method can be used to identify conserved pseudoknotted interactions [56]. Dynamic programming algorithms exist that are able to explicitly predict pseudoknots, albeit at higher computational cost [41,57], and a dynamic programming method performed slightly better in benchmarks of pseudoknot prediction [40]. Therefore, it may be desirable to implement these algorithms in the NCM context.

The CycleFold-TurboFold method presented here is not the only available method to identify conserved non-canonical contacts. Evolutionary information was used recently to infer non-canonical and long-distance contacts using evolutionary coupling with the plmc program, and these contacts were then used to generate structure models [16]. These two methods complement each other; the new method described here provides an important new capability because it does not require a fixed input alignment, and can therefore address the difficulty of aligning RNA sequences. This property is useful for prediction of structures from new families where the exact alignment may be unclear. However, the evolutionary couplings method has the important advantage that it can gracefully consider both pseudoknotted and non-pseudoknotted interactions, which is more difficult using dynamic programming methods. In the small benchmark shown here, it was shown that CycleFold might offer improved accuracy over the evolutionary coupling method; however, in practice, these methods could be used together when performing real structure modeling.

The method described here could be incorporated into tertiary structure modeling efforts in two obvious ways. In the first, non-canonical pairs predicted with CycleFold could be used as an explicit constraint on the search space considered by the modeling program, in much the same way as the canonical secondary structure is currently used. Alternatively, predicted non-canonical pairs could be used in order to help identify RNA folding modules, which typically have a set of known canonical and non-canonical interactions. Additionally, the partition function paradigm would also allow an exact calculation of the probability that a module occurs [58]. Use of identified RNA modules could be used to help understand the function of the RNA, to identify small molecules to target the RNA, or to provide a set of constraints for structure modeling.

In general, predicting only probable pairs or predicting conserved pairs with TurboFold increases the PPV, at a cost of sensitivity. For structure modeling that directly uses these constraints, this is a desirable compromise. Modeling software might yet identify a correct pair if that pair is missing from the input of restraints, but the presence of an incorrect pair would generate an incorrect pair in the tertiary structure, and also likely compromise the accuracy of the model. Accurate predictions of extended secondary structure as provided by this method could prove useful as additional modeling constraints for any tertiary structure modeling program.

The CycleFold program is free software available under the GNU Public License, and it is now part of the RNAstructure software package. Source code, compiled executables, and documentation are available at https://rna.urmc.rochester.edu.

## Materials and methods

### Benchmarks of structure prediction performance

A pair in the predicted structure or in the target structure was considered “canonical” if it was between the nucleotides A-U, G-C, or G-U, and the pairing nucleotides were separated by at least 3 nt in the sequence, which is necessary for the backbone to form the canonical pairing geometry. All other pairs were considered “non-canonical.” Predictions were scored by the sensitivity, which is the fraction of pairs in the accepted structure that were predicted, and the positive predictive value, or PPV, which is the fraction of predicted pairs that were correct. Base pairs were considered to be correct if they are predicted within one nucleotide for one index. That is, if the accepted structure contains a pair at index (i,j), a predicted pair at (i,j), (i+1,j), (i-1,j), (i,j+1), and (i,j-1) is scored as a correct prediction [59]. This is permitted because these alternative pairs are thermodynamically accessible according to solution NMR experiments, which show sampling of alternative base pairing of this kind [60,61], and optical melting experiments of bulges, which are consistent with multiple pairing states [62]. Crystal structures, to which the predictions are compared, generally capture only one pairing state, and therefore requiring exact matches the crystal structure would not be a good assessment of performance.

The complete set of benchmark accuracies for each method on each sequence is provided as a comma-delimited Supplementary Data file (CycleFold_benchmark_accuracy_supplementary-dataset.csv).

### Benchmark of TurboFold and plmc performance

PDB structures 4OJI, 4PQV, and 2XXA were used for the benchmark of TurboFold. Base pairs were extracted with 3DNA-DSSR version 1.1.2 [63]. Homologous sequences for the Twister ribozyme and the Dengue sequence were provided in the publications describing their structures. SRP sequences were acquired from the Signal Recognition Particle Database [64]. From each set of sequences, 9 were chosen randomly to be used by TurboFold. CycleFold was run with default settings. For prediction using plmc, all available aligned sequences were used as input, and the software was run with the recommended settings for RNA.

### Statistical significance of benchmark results

PPV and sensitivity were compared using a paired, two-tailed t-test (ttest_rel function from the SciPy library [65]). For the comparison of PPV, sequences were excluded from the test if either method predicted no pairs. For all t-tests, the type I error rate, α, was set to 0.05. For the comparison between CycleFold and plmc, which both predict pairs associated with a measure of confidence in the predicted pair, the best values of PPV and sensitivity were compared for each sequence.

### Nucleotide Cyclic Motifs

**Fig 6:**
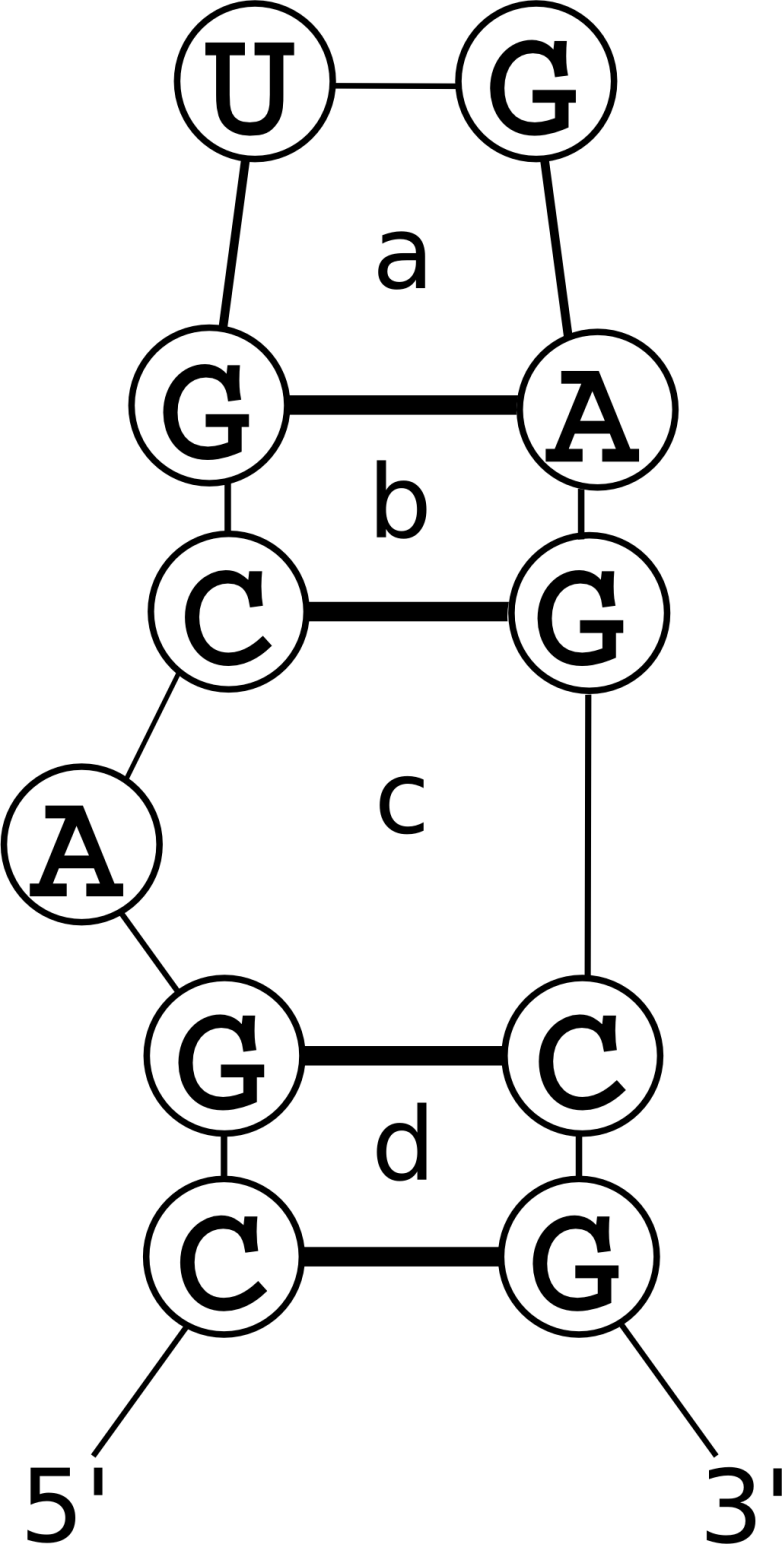
An example of a pseudo-energy calculation using the NCM model. ΔG_junction_ is evaluated for each pair of NCMs that share an edge, i.e. the ones that have an overlapping base pair. This term depends on the identities of the two NCMs (that is, the length of the 5’ and 3’ cycles for a double-stranded NCM, or the total length for a single-stranded NCM), and the nucleotides in the common base pair. This term is evaluated for the junction of NCM a with NCM b, NCM b with NCM c, and NCM c with NCM d.

The NCM model considers an RNA as a graph where the vertices are nucleotides and the edges are either covalent attachments through the backbone or base-pairing interactions, which may be canonical or non-canonical [12]. A Nucleotide Cyclic Motif, or NCM, is a cycle in the graph that contains either one or two edges that correspond to base pairs. As in MC-Fold, a nucleotide may be involved in zero or one base pair; the model does not take into account the possibility of base triples. NCMs with one pair correspond to hairpin loops in RNA structure, while NCMs with two pairs correspond to base pair stacks, bulge loops, or internal loops (Fig 6). Cycles with more than two pairs, which correspond to multibranch loops, are not scored in this model, although the original MC-Fold has an ad-hoc formulation for coaxial stacks, where base pairs from two helices in a multibranch loop can form energetically stable stacking interactions. This was not implemented in CycleFold (or MCFold-DP [33]).

### The NCM scoring model

In the NCM model, the pseudo-free energy of a particular structure is given by

$$\Delta G_{structure}=\underset{m\in ncms}{\sum }\Delta G_{formation}(m,sequence_{m})+\underset{u\in junctions}{\sum }\Delta G_{junction}(u,pair_{u})$$

where *ncms* is the set of all the NCMs in the structure, and *junctions* is the set of all junctions of NCMs in the structure such that two NCMs share a base pair. ΔG_formation_ for an NCM is a function of the identity and the sequence of the NCM, and ΔG_junction_ is a function of the identities of the two NCMs and the sequence of the base pair they share. The NCM model parameters were learned from a set of structures derived from the Protein Data Bank and converted into the pseudo-energies that are tabulated in the NCM data tables [12].

### Example calculation

Fig 6 shows an example of a pseudo-energy calculation using NCMs. This structure contains four NCMs, labelled a-d. NCMs are identified by the number of strands involved in the NCM and by their length; for example a single-stranded NCM of length 4 (representing a hairpin loop with 2 unpaired nucleotides) is referred to as a “1–4” NCM, while a double stranded NCM with 3 nucleotides on the 5’ side and 4 nucleotides on the 3’ side (representing a 1×2 asymmetric internal loop with 1 unpaired nucleotide on the 5’ strand and two unpaired nucleotides on the 3’ strand) is referred to as a “2-3-4” NCM. The ΔG_formation_ for an NCM depends on its dimensions and its sequence.

Therefore, in total,

$$\Delta G_{structure}=\Delta G_{formation}(NCM\;a)+\Delta G_{formation}(NCM\;b)+\Delta G_{formation}(NCM\;c)+\Delta G_{formation}(NCM\;d)+\Delta G_{junction}(NCM\;a,NCM\;b)+\Delta G_{junction}(NCM\;b,NCM\;c)+\Delta G_{junction}(NCM\;c,NCM\;d)$$

$$=\Delta G_{formation}(1-4,GUGA)+\Delta G_{formation}(2-2-2,CGAG)+\Delta G_{formation}(2-3-2,GACGC)+\Delta G_{formation}(2-2-2,CGCG)+\Delta G_{junction}(1-4,2-2-2,GA)+\Delta G_{junction}(2-2-2,2-3-2,CG)+\Delta G_{junction}(2-3-2,2-2-2,GC)$$

=−0.978−1.5130−2.064−1.519−0.3396−0.3148−0.2134=−6.9418

This pseudo-free energy score is provided in units of kcal/mol. However, they do not reflect physical properties of the sequence, rather the log of the frequency with which these NCMs are observed in the training set. The parameters used in CycleFold are taken from MC-Fold-DP [33], which derived its parameters from tables from MC-Fold [12].

#### The partition function

The partition function, *Q*, is the sum of the equilibrium constants of all structures and it describes the thermodynamic ensemble. It is given by

$$Q=\overset{n}{\underset{s=1}{\sum }}e^{-∆G_{s}/RT}$$

where *n* is the number of possible structures of the system, ΔG_s_ is the Gibbs free energy change of folding to structure *s*, *R* is the universal gas constant, and *T* is the absolute temperature (310.15 K in this work). At equilibrium, the probability of structure, *s’*, is given by

$$P(s\prime )=\frac{e^{-∆G_{s\prime }/RT}}{Q}$$

Therefore, the partition function quantifies the entire thermodynamic ensemble in a way that the probability of any state of the system can be calculated using the Gibbs free energy change of that state.

The probability *P*_*i*,*j*_ of a pair between nucleotides *i* and *j* is given by

$$P_{ij}=\underset{e_{ij}}{\sum }P(k)=\underset{s^{\prime \prime }\in b_{ij}}{\sum }\frac{e^{-∆G_{k}/RT}}{Q}$$

where *b*_*ij*_ is the set of structures in which *i* and *j* are paired. In other words, the probability that *i* and *j* are paired is the sum of the probabilities of each structure that contains a pair between *i* and *j*. This probability can be calculated efficiently without enumerating all the structures in *k* by using intermediate results from the calculation of the full partition function, as described below.

### Calculation of the partition function using the NCM model

**Fig 7:**
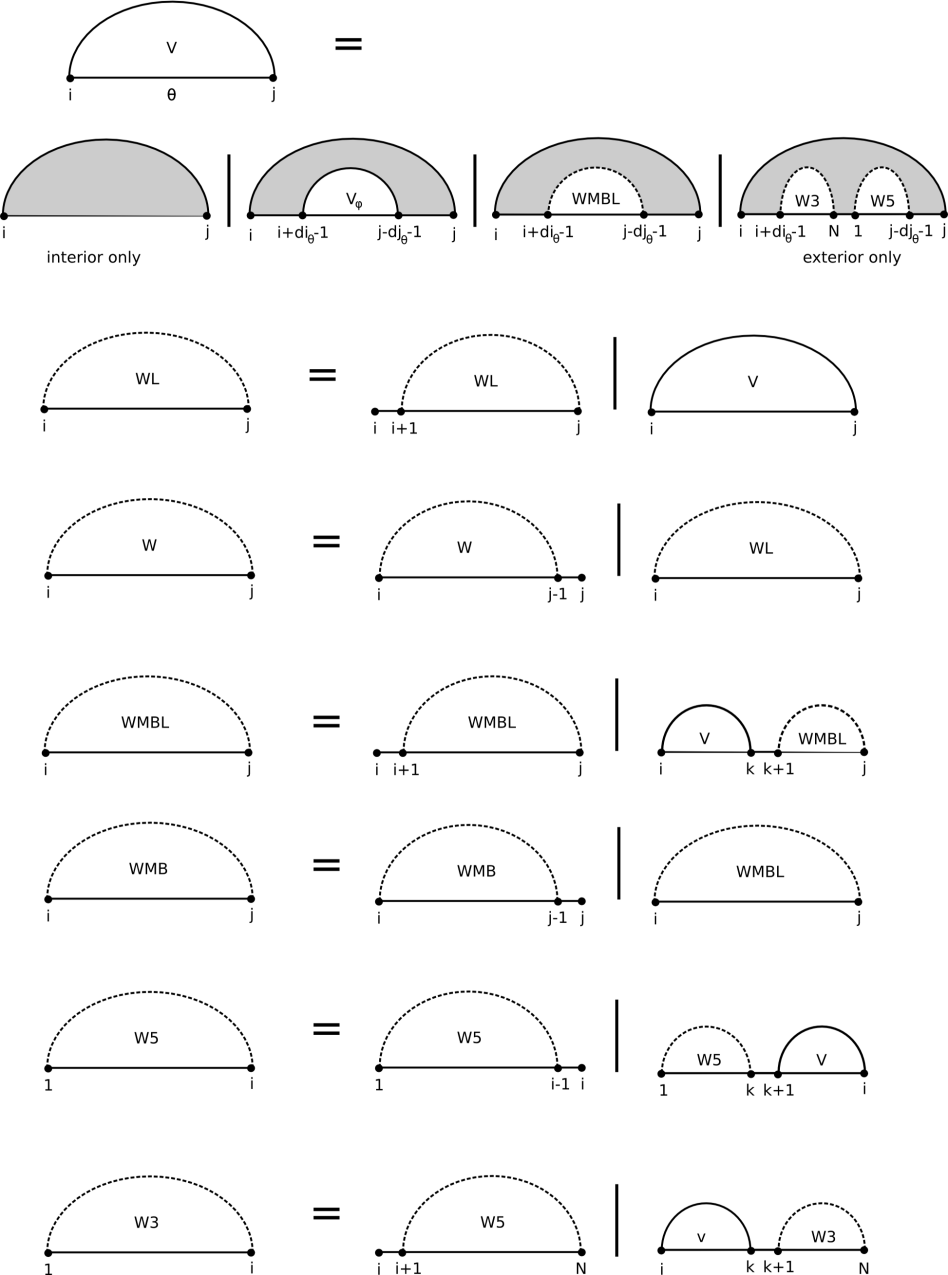
A recursion diagram [41] illustrating the NCM partition function algorithm. Filled regions indicate terms that are being added to the partition function, and empty regions indicate results that were previously calculated. Solid lines indicate nucleotides that must be paired, while dotted lines indicate nucleotides that may or may not be paired.

The partition function using the NCM model can be calculated efficiently using a dynamic programming algorithm (Fig 7). The algorithm scales *O*(N^3^), where N is the length of the sequence, and this is the same scaling as MC-Fold-DP and the free energy minimization mode of CycleFold. Time benchmarks of the partition function (and the TurboFold extension), compared to the partition function across canonical pairs is provided in S9 Table. This algorithm stores intermediate results in seven tables. The first, *V*, is a table of size N×N×γ, where γ is the number of distinct NCM types. *V(i*,*j*,*θ)* stores the partition function for the fragment from *i* to *j* closed by an NCM of type *θ*. Four N×N tables, *W*, *WL*, *WMB*, *and WMBL*, store components of multibranched structures [36]. *W* and *WL* store the partition function for the fragment from i to j where i and j are not required to be paired to one another, and the fragment contains one helix. *WL* has the additional restriction that the nucleotide at j is required to be one of the closing base pairs of the helix. *WMB* and *WMBL* store the partition function for the fragment from *i* to *j* containing two or more helices, and *WMBL* has the additional restriction that *j* is one of the closing nucleotides of the 5’-most helix. The final two arrays, *W5* and *W3*, are tables of size N that store the partition function for the fragment from 1 to i and i to N, respectively. At the end of the calculation, W5(N) = W3(1) = Q. In *V(i*,*j*,*θ)*, *W(i*,*j)*, *WL(i*,*j)*, *WMB(i*,*j)*, and *WMBL(i*,*j)*, entries where i<j refer to interior fragments, while entries where i>j refer to exterior fragments, containing the ends of the sequence. Prior to the calculation, all array values are initialized to 0 except for W5(1) and W3(N), which are initialized to 1 to represent the equilibrium constant of the completely unfolded state (which is the reference state with folding free energy of zero).

The *V* table entry is the sum of the following terms:

$$V(i,j,\theta )=V_{hairpin}(i,j,\theta )+V_{stack}(i,j,\theta )+V_{multibranch}(i,j,\theta )$$

If this is an exterior fragment, an additional term V_exterior_ replaces *V*_*hairpin*_. These components are calculated as follows.

$$V_{hairpin}(i,j,\theta )=e^{{-∆G}_{formation}(\theta )/RT}$$

where ΔG_hairpin_ depends on the sequence of the fragment and is tabulated as part of the NCM parameters.

$$V_{stack}(i,j,\theta )=\underset{\varphi \in \phi }{\sum }e^{{-∆G}_{formation}(\theta )/RT}\times e^{{-∆G}_{junction}(\theta ,\varphi )/RT}\times V(i+di_{\theta }-1,j-dj_{\theta }+1,\varphi )$$

where ϕ is the set of NCMs that form valid structures when they abut on NCM *θ* at position *i*,*j*, and *di*_θ_ and *dj*_θ_ are the length of θ on the 5’ and 3’ sides, respectively.

$$V_{multibranch}(i,j,\theta )=e^{{-∆G}_{formation}(\theta )/RT}\times WMBL(i+di_{\theta }-1,j-dj_{\theta }+1)$$

If *i*,*j* forms an exterior fragment, V_exterior_ is calculated:

$$V_{exterior}(i,j,\theta )=e^{{-∆G}_{formation}(\theta )/RT}\times W5(j-dj_{\theta }+1)\times W3(i+di_{\theta }+1)$$

The tables to save components of a multibranch loop are calculated as follows:

$$WL(i,j)=WL(i+1,j)+\underset{\varphi \in \phi }{\sum }V(i,j,\varphi )$$

W(i,j)=WL(i,j)+W(i,j−1)

$$WMBL(i,j)=WMBL(i+1,j)+\underset{i<k<j}{\sum }\underset{\varphi \in \phi }{\sum }V(i,k,\varphi )\times [WMBL(k+1,j)+WL(k+1,j)]$$

WMB(i,j)=WMBL(i,j)+WMB(i,j−1)

Finally, the 1-dimensional tables *W5* and *W3* are calculated as follows:

$$W5=W5(i-1)+\underset{1<k<i}{\sum }\underset{\varphi \in \phi }{\sum }W5(k)\times V(k+1,i,\varphi )$$

$$W3=W3(i+1)+\underset{i<k<N}{\sum }\underset{\varphi \in \phi }{\sum }V(i,k-1,\varphi )\times W3(k)$$

Some structural configurations, such as large bulge loops, cannot be captured by the NCM model because there is no appropriately-sized NCM. In order to allow the consideration of these structures, an additional contribution to the partition function from these “long loops” may be used.

$$V_{LL}(i,j,\theta )=\underset{i\prime }{\sum }\underset{j\prime }{\sum }\underset{\varphi \in \phi }{\sum }V(i\prime ,j\prime ,\varphi )$$

where *i* and *j* are nucleotide indices chosen so that *i* < *i’* < *j’* < *j* and there is no valid NCM containing the pairs *i-j* or *i-j* and *i’-j’*. CycleFold does not perform this calculation by default, but it may be enabled by the user. This is important when pairing constraints are used, where the constraints could force the prediction to use a “long loop.”

#### Calculation of base pairing probabilities

Once the dynamic programming tables have been filled, the probability for a base pair in a structure is given by

$$P(i,j)=\frac{1}{W3(1)}\underset{\varphi ,\gamma \in \phi }{\sum }V(i,j,\varphi )\times V(j,i,\gamma )\times e^{{-∆G}_{junction}(\varphi ,\gamma )/RT}$$

where ϕ is the set of pairs of NCMs φ,γ that can form a junction sharing a pair at position *i*,*j*.

### Ambiguity testing

The partition function recursions need to consider each valid NCM structure once and only once. The recursions were designed to non-redundantly consider each structure, where the multibranch loop recursions were taken from the prior implementation for canonical base pairs [36].

To test for redundancy, fuzz testing was performed using a set of 22,000 random sequences of length 75. Canonical base pairs were predicted for each sequence using stochastic sampling [66] in RNAstructure [67]. Then, the structures were predicted using CycleFold, where the canonical pairs were constrained to occur. The canonical base pair probabilities were then verified to exactly 1, within tolerance for machine precision, for all sequences. This empirically supports that the recursions are non-redundant because redundancy could result in probabilities smaller or larger than 1 for the constrained pairs.

### Adaptation of recursions to predict minimum free energy structure

These recurrence equations can be adapted to find the minimum-free energy structure by replacing each sum operation with a min operation and each product operation with a sum. In the CycleFold, a generic set of recursions that are used by both algorithms is implemented with C++ templates. The templated energy functions can be instantiated with integer types representing free energy changes (where free energies are multiplied by 10^5^ and stored to 10^−5^ kcal/mol precision), in which case mins of sums are calculated, yielding a minimum free energy. If the templates are instantiated with floating-point types, representing equilibrium constants, then sums of products are calculated, yielding a partition function.

### TurboFold algorithm

Consensus pair probabilities for multiple homologous sequences were determined using the TurboFold algorithm [47]. A TurboFold calculation uses tables of pairing probabilities from a partition function calculation on each sequence and a probabilistic alignment between each pair of sequences calculated with a pair Hidden Markov Model. The pairing probabilities are updated in each of multiple iterations of the algorithm. TurboFold follows the intuition that, if nucleotides *i* and *j* are likely to be paired in an RNA sequence *A*, and nucleotides *i* and *j* in sequence *A* are believed to be aligned to nucleotides *i’* and *j’* in a related sequence *B*, then this is evidence that *i’* and *j’* form a base pair. A table of pairing proclivities containing information about the structure of sequence *B* inferred from sequence *A* is called the *E*_*AB*_, the extrinsic information about *B* from *A*. That is,

$$E_{AB}(i\prime ,j\prime )=\underset{1\leq i\leq N_{A}}{\sum }\underset{1\leq j\leq N_{A}}{\sum }P_{A}(i,j)\times \Pi_{AB}(i,i^{\prime })\times \Pi_{AB}(j,j^{\prime })$$

where *N*_*A*_ is the length of sequence *A*, *π*_*AB*_ is a table containing the pair HMM alignment between *A* and *B*, and *P*_*A*_ is the table of pairing probabilities for sequence *A*. An analogous calculation is used to calculate *E*_*BA*_, the extrinsic information about *A* from *B*.

The extrinsic information for each related sequence is summed and normalized by the largest entry. This combined extrinsic information *E* can be incorporated into the partition function algorithm by altering the calculation of *V*(*i*,*j*,*θ*):

$$V(i,j,\theta )=[V_{hairpin}(i,j,\theta )+V_{stack}(i,j,\theta )+V_{multibranch}(i,j,\theta )]\times \rho e^{E(i,j)}$$

where *ρ* is a fitted parameter that quantifies the weight associated with the extrinsic information. In the implementation of CycleFold, the previously determined weight of 0.3 was used. Because the extrinsic information is normalized to a value between 0 and 1, this modification penalizes structures containing pairs inconsistent with the extrinsic information by weighting them less strongly in the partition function.

In the TurboFold calculation, pairwise probabilistic alignments are calculated between each structure using a pair hidden Markov model (HMM), and the partition function for each sequence is calculated. Then, the extrinsic information and the partition function for each sequence is iteratively re-calculated, first updating the partition function using the extrinsic information, then updating the extrinsic information with the partition function-determined pair probabilities.

The RNAstructure software package was updated to include a generic implementation of the TurboFold algorithm that is independent of the actual partition function implementation, and can iteratively re-estimate pair probabilities using any user-provided partition function. This could be useful for, as an example, custom partition dynamic programming algorithms that incorporate knowledge of the structure family of interest [68,69].

## Supporting information

S1 Fig Native structures from (A) nuclease-resistant sequence from a Murray Valley Encephalitis virus 3’ UTR (PDB 4PQV), (B) *D*. *radiodurans* SRP hairpin domain (PDB 2XXA), and (C) twister ribozyme from *Oryza sativa* (PDB 4OIJ). Base pairs are drawn with black lines for canonical pairs and red lines for non-canonical pairs. Base pairs were found from the coordinates using 3DNA-DSSR version 1.1.2, all cis-Watson-Watson pairs are called canonical.
(PDF)

S1 Table PDB codes of sequences used from RNAstrand in benchmark. (PDF)

S2 Table Performance of structure prediction methods. (PDF)

S3 Table Statistical comparison for prediction of canonical pairs with energy minimization algorithm. (PDF)

S4 Table Statistical comparison of CycleFold partition function against energy minimization algorithms for canonical pairs. (PDF)

S5 Table Statistical comparison for prediction of non-canonical pairs with energy minimization algorithm. (PDF)

S6 Table Statistical comparison of CycleFold partition function against energy minimization algorithms for non-canonical pairs. (PDF)

S7 Table Statistical comparison of CycleFold (MFE) partition function with canonical constraints against energy minimization algorithms for non-canonical pairs. (PDF)

S8 Table Statistical comparison of performance between CycleFold-TurboFold and plmc. (PDF)

S9 Table Time benchmarks. (PDF)

S1 Data Information about structure prediction accuracy of each sequence when using CycleFold, Fold, MC-Fold, and MC-Fold-DP. (CSV)
