# Supplementary material for: Base pair probability estimates improve the prediction accuracy of RNA non-canonical base pairs
Source: PLoS Comput Biol. 2017 Nov 6;13(11):e1005827. doi: 10.1371/journal.pcbi.1005827 (PMC5690697; doi:10.1371/journal.pcbi.1005827)
Supplement: S1 Fig — Native structures from (A) nuclease-resistant sequence from a Murray Valley Encephalitis virus 3’ UTR (PDB 4PQV), (B) D. radiodurans SRP hairpin domain (PDB 2XXA), and (C) twister ribozyme from Oryza sativa (PDB 4OIJ). Base pairs are drawn with black lines for canonical pairs and red lines for non-canonical pairs. Base pairs were found from the coordinates using 3DNA-DSSR version 1.1.2, all cis-Watson-Watson pairs are called canonical. (PDF) [file pcbi.1005827.s001.pdf]

Supporting Figure 1A. Nuclease-resistant sequence from a Murray Valley Encephalitis virus 3' UTR.

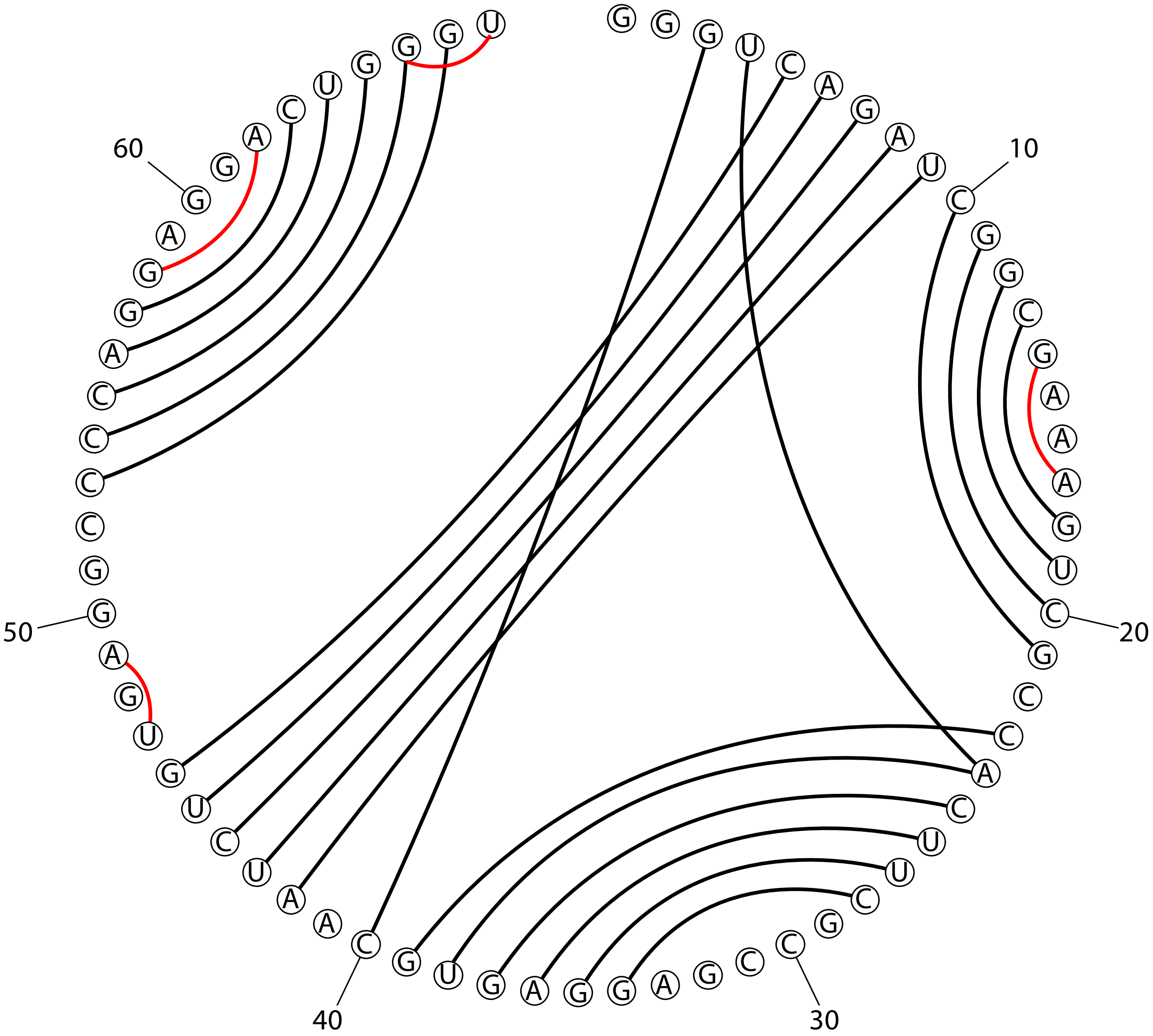

Supporting Figure 1B.

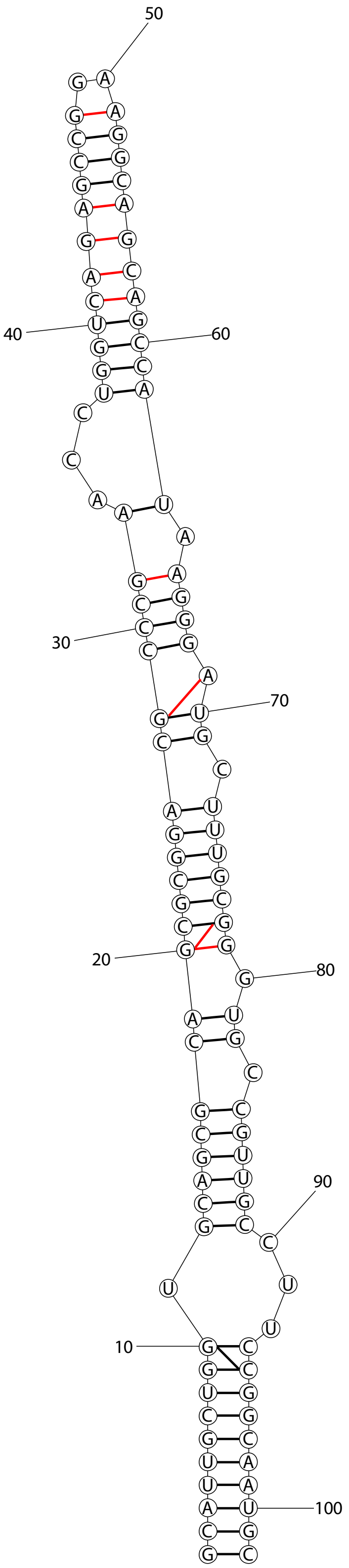

Supporting Figure 1C. Twister ribozyme from *Oryza sativa.*

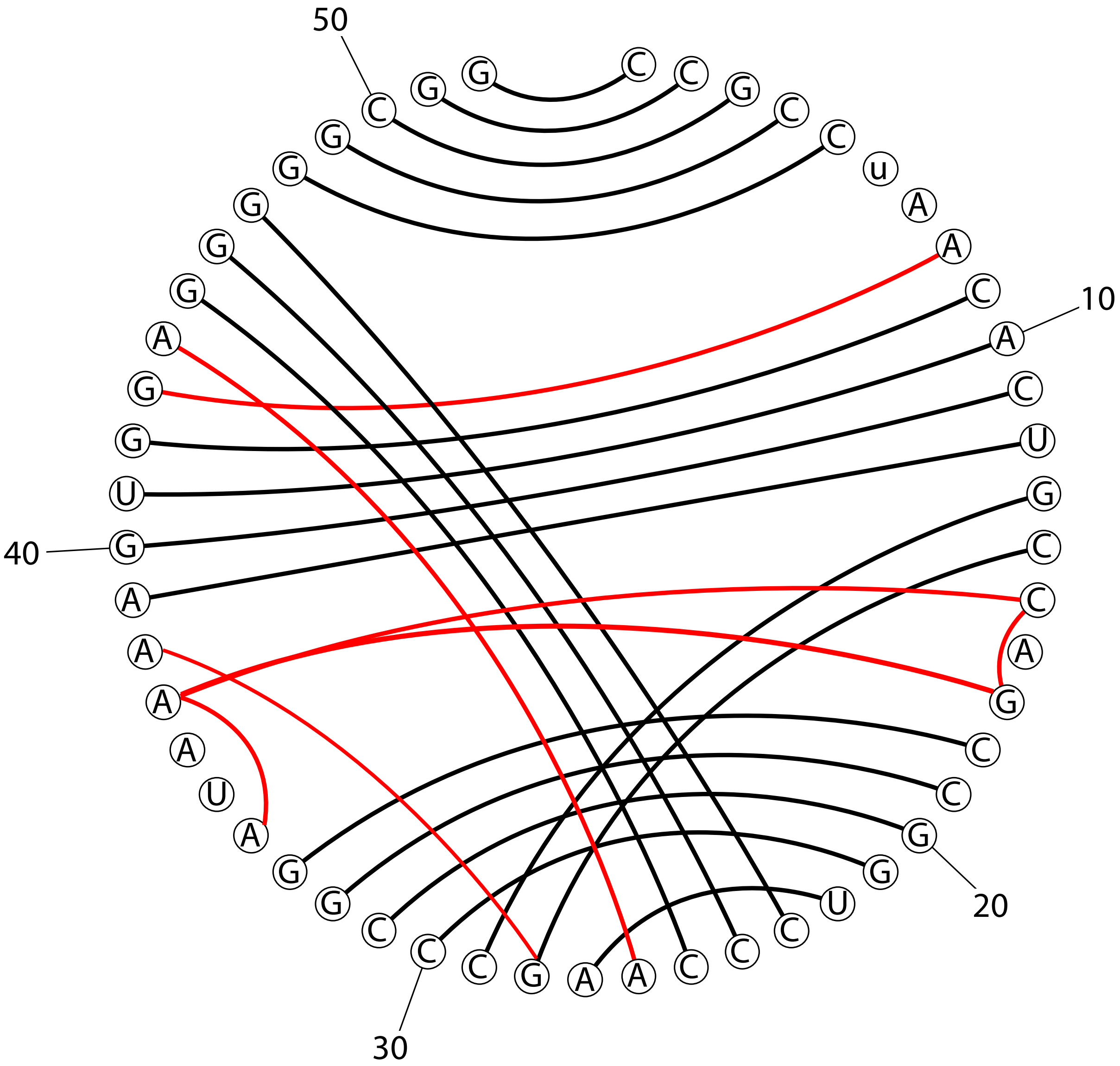

Supporting Figure 1. Native structures from (A) nuclease-resistant sequence from a Murray Valley Encephalitis virus 3’ UTR (PDB 4PQV), (B) *D. radiodurans* SRP hairpin domain (PDB 2XXA), and (C) twister ribozyme from *Oryza sativa* (PDB 4OIJ). Base pairs are drawn with black lines for canonical pairs and red lines for non-canonical pairs. Base pairs were found from the coordinates using 3DNA-DSSR version 1.1.2, all cis-Watson-Watson pairs are called canonical.
