# Supplementary material for: Base pair probability estimates improve the prediction accuracy of RNA non-canonical base pairs
Source: PLoS Comput Biol. 2017 Nov 6;13(11):e1005827. doi: 10.1371/journal.pcbi.1005827 (PMC5690697; doi:10.1371/journal.pcbi.1005827)
Supplement: S1 Table — (PDF) [file pcbi.1005827.s002.pdf]

Supporting Table S1: PDB codes of sequences used from RNAstrand in benchmark.

165d	1f7f	1s34	2azx	2gio	2izm	2qh2
1a1t	1f7g	1scl	2b2d	2gip	2izn	2qh3
1a3m	1f7h	1slo	2b63	2gjw	2jpp	2qh4
1a4d	1f7i	1sy4	2b7g	2grw	2jr4	2qux
1a9l	1fje	1tbk	2cd1	2gv3	2jrg	2r92
1aju	1fyp	1tfy	2cd3	2gv4	2jrq	2r93
1akx	1i4c	1u1y	2cd5	2gvo	2jse	2u2a
1anr	1ie2	1urn	2cd6	2h2x	2jsg	3bsn
1aqo	1jbs	1uts	2dd1	2hem	2jtp	3bso
1arj	1jp0	1uui	2dd2	2hgh	2juk	387d
1biv	1jwc	1wtt	2dd3	2hns	2jwv	
1bz3	1kka	1xst	2der	2hoj	2ldz	
1cql	1lc6	1xsu	2dr8	2hua	2nok	
1ebq	1mnb	1ylg	2du6	2hvy	2nue	
1ebs	1mv2	1yn2	2ese	2hw8	2o33	
1eht	1p6v	1yng	2evy	2i7e	2o81	
1ei2	1pbr	1ysh	2f87	2i7z	2o83	
1eor	1q2r	1zbh	2f8s	2i82	2oe5	
1esy	1qcu	1zbn	2f8t	2i91	2oiu	
1etf	1qfq	28sr	2fdt	2ipy	2oj7	
1etg	1qwa	2a9l	2fey	2irn	2oj8	
1f1t	1qzw	2aht	2fy1	2iro	2ozb	
1f78	1rht	2ake	2g1g	2ixy	2pcv	
1f79	1rmn	2ann	2g91	2iz9	2pjp	
