# Supplementary material for: Base pair probability estimates improve the prediction accuracy of RNA non-canonical base pairs
Source: PLoS Comput Biol. 2017 Nov 6;13(11):e1005827. doi: 10.1371/journal.pcbi.1005827 (PMC5690697; doi:10.1371/journal.pcbi.1005827)
Supplement: S2 Table — (PDF) [file pcbi.1005827.s003.pdf]

Supporting Table S2: Performance of structure prediction methods.

Supporting Table S2A: Mean PPV and sensitivity for CycleFold. The MFE performance has a single row. The partition function performance is shown as a function of probability threshold. Constrained indicates that the known canonical pairs were constrained.

Threshold	CycleFold canonical PPV	CycleFold canonical sens.	CycleFold constrained non-canonical PPV	CycleFold constrained non-canonical sens.	CycleFold non-canonical PPV	CycleFold non-canonical sens.
MFE	0.819	0.863	0.592	0.768	0.470	0.640
0.1	0.769	1.012	0.468	1.076	0.426	1.040
0.2	0.803	0.955	0.522	0.937	0.519	0.913
0.3	0.812	0.912	0.561	0.936	0.521	0.760
0.4	0.824	0.876	0.642	0.851	0.563	0.623
0.5	0.836	0.818	0.693	0.762	0.625	0.470
0.6	0.850	0.789	0.717	0.738	0.639	0.409
0.7	0.880	0.746	0.708	0.668	0.653	0.304
0.8	0.899	0.696	0.682	0.477	0.650	0.239
0.85	0.909	0.643	0.738	0.427	0.647	0.174
0.9	0.918	0.528	0.713	0.344	0.667	0.087
0.95	0.954	0.401	0.889	0.297	0.750	0.051
0.99	0.957	0.051	1.000	0.024		0.000

Supporting Table S2B. Mean PPV and sensitivity for Fold.

Fold canonical PPV	Fold canonical sens.
0.834	0.803

Supporting Table S2C. Mean PPV and sensitivity of MC-Fold.

MC-Fold canonical PPV	MC-Fold canonical sens.	MC-Fold non-canonical PPV	MC-Fold non-canonical sens.
0.809	0.866	0.478	0.623

Supporting Table S2D. Mean PPV and sensitivity of MC-Fold-DP.

MC-Fold-DP canonical PPV	MC-Fold-DP canonical sens.	MC-Fold-DP non-canonical PPV	MC-Fold-DP non-canonical sens.
0.825	0.816	0.448	0.773
