# Supplementary material for: Base pair probability estimates improve the prediction accuracy of RNA non-canonical base pairs
Source: PLoS Comput Biol. 2017 Nov 6;13(11):e1005827. doi: 10.1371/journal.pcbi.1005827 (PMC5690697; doi:10.1371/journal.pcbi.1005827)
Supplement: S3 Table — (PDF) [file pcbi.1005827.s004.pdf]

Supporting Table S3: Statistical comparison for prediction of canonical pairs with energy minimization algorithm. If $p < 0.05$, the name of the program with significantly higher performance is provided.

program 1	program 2	metric	Significantly better performer	p value
Fold	MC-Fold	PPV	Fold	0.013
Fold	MC-Fold	sens.	none	0.563
Fold	MC-Fold-DP	PPV	none	0.698
Fold	MC-Fold-DP	sens.	none	0.680
Fold	CycleFold	PPV	Fold	0.032
Fold	CycleFold	sens.	none	0.721
MC-Fold	MC-Fold-DP	PPV	none	0.438
MC-Fold	MC-Fold-DP	sens.	MC-Fold	0.016
MC-Fold	CycleFold	PPV	none	0.507
MC-Fold	CycleFold	sens.	none	0.677
MC-Fold-DP	CycleFold	PPV	none	0.596
MC-Fold-DP	CycleFold	sens.	CycleFold	0.000
