# Supplementary material for: Base pair probability estimates improve the prediction accuracy of RNA non-canonical base pairs
Source: PLoS Comput Biol. 2017 Nov 6;13(11):e1005827. doi: 10.1371/journal.pcbi.1005827 (PMC5690697; doi:10.1371/journal.pcbi.1005827)
Supplement: S4 Table — (PDF) [file pcbi.1005827.s005.pdf]

Supporting Table S4: Statistical comparison of CycleFold partition function against energy minimization algorithms for canonical pairs. If $p < 0.05$, the name of the program with significantly higher performance is provided.

program 1	program 2	metric	Significantly better performer	p value
Fold	CycleFold_thresholded	PPV(threshold 0.5)	none	1.026E-01
Fold	CycleFold_thresholded	sens.(threshold 0.5)	none	2.887E-01
Fold	CycleFold_thresholded	PPV(threshold 0.6)	none	3.182E-01
Fold	CycleFold_thresholded	sens.(threshold 0.6)	Fold	4.357E-02
Fold	CycleFold_thresholded	PPV(threshold 0.7)	none	9.952E-01
Fold	CycleFold_thresholded	sens.(threshold 0.7)	Fold	1.172E-03
Fold	CycleFold_thresholded	PPV(threshold 0.8)	none	4.172E-01
Fold	CycleFold_thresholded	sens.(threshold 0.8)	Fold	1.727E-06
Fold	CycleFold_thresholded	PPV(threshold 0.85)	none	5.663E-01
Fold	CycleFold_thresholded	sens.(threshold 0.85)	Fold	1.508E-10
Fold	CycleFold_thresholded	PPV(threshold 0.9)	none	2.457E-01
Fold	CycleFold_thresholded	sens.(threshold 0.9)	Fold	1.499E-17
Fold	CycleFold_thresholded	PPV(threshold 0.95)	none	1.815E-01
Fold	CycleFold_thresholded	sens.(threshold 0.95)	Fold	2.932E-26
Fold	CycleFold_thresholded	PPV(threshold 0.99)	none	7.489E-01
Fold	CycleFold_thresholded	sens.(threshold 0.99)	Fold	2.179E-60
MC-Fold	CycleFold_thresholded	PPV(threshold 0.5)	CycleFold_thresholded	8.110E-03
MC-Fold	CycleFold_thresholded	sens.(threshold 0.5)	MC-Fold	1.496E-02
MC-Fold	CycleFold_thresholded	PPV(threshold 0.6)	CycleFold_thresholded	4.516E-03
MC-Fold	CycleFold_thresholded	sens.(threshold 0.6)	MC-Fold	1.019E-04
MC-Fold	CycleFold_thresholded	PPV(threshold 0.7)	CycleFold_thresholded	4.421E-04
MC-Fold	CycleFold_thresholded	sens.(threshold 0.7)	MC-Fold	2.848E-08
MC-Fold	CycleFold_thresholded	PPV(threshold 0.8)	CycleFold_thresholded	1.870E-05

MC-Fold	CycleFold_thresholded	sens.(threshold 0.8)	MC-Fold	1.378E-13
MC-Fold	CycleFold_thresholded	PPV(threshold 0.85)	CycleFold_thresholded	1.283E-05
MC-Fold	CycleFold_thresholded	sens.(threshold 0.85)	MC-Fold	1.122E-18
MC-Fold	CycleFold_thresholded	PPV(threshold 0.9)	CycleFold_thresholded	2.047E-05
MC-Fold	CycleFold_thresholded	sens.(threshold 0.9)	MC-Fold	1.554E-22
MC-Fold	CycleFold_thresholded	PPV(threshold 0.95)	CycleFold_thresholded	2.376E-06
MC-Fold	CycleFold_thresholded	sens.(threshold 0.95)	MC-Fold	4.063E-29
MC-Fold	CycleFold_thresholded	PPV(threshold 0.99)	CycleFold_thresholded	9.419E-04
MC-Fold	CycleFold_thresholded	sens.(threshold 0.99)	MC-Fold	2.112E-59
MC-Fold-DP	CycleFold_thresholded	PPV(threshold 0.5)	none	2.697E-01
MC-Fold-DP	CycleFold_thresholded	sens.(threshold 0.5)	none	9.004E-01
MC-Fold-DP	CycleFold_thresholded	PPV(threshold 0.6)	none	1.627E-01
MC-Fold-DP	CycleFold_thresholded	sens.(threshold 0.6)	none	1.816E-01
MC-Fold-DP	CycleFold_thresholded	PPV(threshold 0.7)	CycleFold_thresholded	1.896E-02
MC-Fold-DP	CycleFold_thresholded	sens.(threshold 0.7)	MC-Fold-DP	2.699E-03
MC-Fold-DP	CycleFold_thresholded	PPV(threshold 0.8)	CycleFold_thresholded	1.805E-02
MC-Fold-DP	CycleFold_thresholded	sens.(threshold 0.8)	MC-Fold-DP	1.443E-05
MC-Fold-DP	CycleFold_thresholded	PPV(threshold 0.85)	CycleFold_thresholded	1.634E-02
MC-Fold-DP	CycleFold_thresholded	sens.(threshold 0.85)	MC-Fold-DP	1.200E-09
MC-Fold-DP	CycleFold_thresholded	PPV(threshold 0.9)	none	6.162E-02
MC-Fold-DP	CycleFold_thresholded	sens.(threshold 0.9)	MC-Fold-DP	2.820E-14
MC-Fold-DP	CycleFold_thresholded	PPV(threshold 0.95)	CycleFold_thresholded	6.915E-03
MC-Fold-DP	CycleFold_thresholded	sens.(threshold 0.95)	MC-Fold-DP	4.011E-20
MC-Fold-DP	CycleFold_thresholded	PPV(threshold 0.99)	none	1.104E-01
MC-Fold-DP	CycleFold_thresholded	sens.(threshold 0.99)	MC-Fold-DP	2.877E-43
CycleFold	CycleFold_thresholded	PPV(threshold 0.5)	CycleFold_thresholded	4.782E-03
CycleFold	CycleFold_thresholded	sens.(threshold 0.5)	CycleFold	5.445E-03

CycleFold	CycleFold_thresholded	PPV(threshold 0.6)	CycleFold_thresholded	1.733E-03
CycleFold	CycleFold_thresholded	sens.(threshold 0.6)	CycleFold	2.949E-06
CycleFold	CycleFold_thresholded	PPV(threshold 0.7)	CycleFold_thresholded	7.163E-05
CycleFold	CycleFold_thresholded	sens.(threshold 0.7)	CycleFold	1.131E-09
CycleFold	CycleFold_thresholded	PPV(threshold 0.8)	CycleFold_thresholded	2.726E-07
CycleFold	CycleFold_thresholded	sens.(threshold 0.8)	CycleFold	4.398E-13
CycleFold	CycleFold_thresholded	PPV(threshold 0.85)	CycleFold_thresholded	2.402E-07
CycleFold	CycleFold_thresholded	sens.(threshold 0.85)	CycleFold	1.456E-17
CycleFold	CycleFold_thresholded	PPV(threshold 0.9)	CycleFold_thresholded	1.777E-05
CycleFold	CycleFold_thresholded	sens.(threshold 0.9)	CycleFold	2.948E-22
CycleFold	CycleFold_thresholded	PPV(threshold 0.95)	CycleFold_thresholded	3.914E-06
CycleFold	CycleFold_thresholded	sens.(threshold 0.95)	CycleFold	4.569E-29
CycleFold	CycleFold_thresholded	PPV(threshold 0.99)	CycleFold_thresholded	6.727E-04
CycleFold	CycleFold_thresholded	sens.(threshold 0.99)	CycleFold	2.347E-61
