# Supplementary material for: Base pair probability estimates improve the prediction accuracy of RNA non-canonical base pairs
Source: PLoS Comput Biol. 2017 Nov 6;13(11):e1005827. doi: 10.1371/journal.pcbi.1005827 (PMC5690697; doi:10.1371/journal.pcbi.1005827)
Supplement: S5 Table — (PDF) [file pcbi.1005827.s006.pdf]

Supporting Table S5: Statistical comparison for prediction of non-canonical pairs with energy minimization algorithm. If $p < 0.05$, the name of the program with significantly higher performance is provided.

program 1	program 2	metric	Significantly better performer	p value
MC-Fold	MC-Fold-DP	PPV	none	2.892E-01
MC-Fold	MC-Fold-DP	STY	MC-Fold-DP	3.515E-04
MC-Fold	CycleFold	PPV	none	5.433E-01
MC-Fold	CycleFold	STY	CycleFold	3.515E-04
MC-Fold	CycleFold_constrained	PPV	CycleFold_constrained	5.621E-04
MC-Fold	CycleFold_constrained	STY	CycleFold_constrained	3.515E-04
MC-Fold-DP	CycleFold	PPV	none	8.769E-01
MC-Fold-DP	CycleFold	STY	CycleFold	3.515E-04
MC-Fold-DP	CycleFold_constrained	PPV	CycleFold_constrained	4.565E-02
MC-Fold-DP	CycleFold_constrained	STY	CycleFold_constrained	3.515E-04
CycleFold	CycleFold_constrained	PPV	CycleFold_constrained	4.360E-04
CycleFold	CycleFold_constrained	STY	CycleFold_constrained	3.515E-04
