# Supplementary material for: Base pair probability estimates improve the prediction accuracy of RNA non-canonical base pairs
Source: PLoS Comput Biol. 2017 Nov 6;13(11):e1005827. doi: 10.1371/journal.pcbi.1005827 (PMC5690697; doi:10.1371/journal.pcbi.1005827)
Supplement: S6 Table — (PDF) [file pcbi.1005827.s007.pdf]

Supporting Table S6: Statistical comparison of CycleFold partition function against energy minimization algorithms for non-canonical pairs. If $p < 0.05$, the name of the program with significantly higher performance is provided. For p value, nan (not a number) appears when the PPV was undefined for one or two programs.

program 1	program 2	metric	Significantly better performer	p value
MC-Fold	CycleFold_thresholded	PPV(threshold 0.5)	none	6.368E-02
MC-Fold	CycleFold_thresholded	sens.(threshold 0.5)	MC-Fold	2.435E-02
MC-Fold	CycleFold_thresholded	PPV(threshold 0.6)	none	2.682E-01
MC-Fold	CycleFold_thresholded	sens.(threshold 0.6)	MC-Fold	3.806E-03
MC-Fold	CycleFold_thresholded	PPV(threshold 0.7)	none	5.762E-01
MC-Fold	CycleFold_thresholded	sens.(threshold 0.7)	MC-Fold	5.699E-05
MC-Fold	CycleFold_thresholded	PPV(threshold 0.8)	none	7.481E-01
MC-Fold	CycleFold_thresholded	sens.(threshold 0.8)	MC-Fold	9.125E-07
MC-Fold	CycleFold_thresholded	PPV(threshold 0.85)	none	7.498E-01
MC-Fold	CycleFold_thresholded	sens.(threshold 0.85)	MC-Fold	3.358E-08
MC-Fold	CycleFold_thresholded	PPV(threshold 0.9)	none	6.952E-01
MC-Fold	CycleFold_thresholded	sens.(threshold 0.9)	MC-Fold	9.036E-10
MC-Fold	CycleFold_thresholded	PPV(threshold 0.95)	none	nan
MC-Fold	CycleFold_thresholded	sens.(threshold 0.95)	MC-Fold	8.912E-11
MC-Fold	CycleFold_thresholded	PPV(threshold 0.99)	none	nan
MC-Fold	CycleFold_thresholded	sens.(threshold 0.99)	MC-Fold	5.389E-12
MC-Fold-DP	CycleFold_thresholded	PPV(threshold 0.5)	none	3.415E-01
MC-Fold-DP	CycleFold_thresholded	sens.(threshold 0.5)	MC-Fold-DP	4.405E-05
MC-Fold-DP	CycleFold_thresholded	PPV(threshold 0.6)	none	9.514E-01
MC-Fold-DP	CycleFold_thresholded	sens.(threshold 0.6)	MC-Fold-DP	2.296E-06
MC-Fold-DP	CycleFold_thresholded	PPV(threshold 0.7)	none	6.234E-01
MC-Fold-DP	CycleFold_thresholded	sens.(threshold 0.7)	MC-Fold-DP	3.837E-08
MC-Fold-DP	CycleFold_thresholded	PPV(threshold 0.8)	none	6.215E-01

MC-Fold-DP	CycleFold_thresholded	sens.(threshold 0.8)	MC-Fold-DP	1.682E-09
MC-Fold-DP	CycleFold_thresholded	PPV(threshold 0.85)	none	6.237E-01
MC-Fold-DP	CycleFold_thresholded	sens.(threshold 0.85)	MC-Fold-DP	2.137E-11
MC-Fold-DP	CycleFold_thresholded	PPV(threshold 0.9)	none	9.164E-01
MC-Fold-DP	CycleFold_thresholded	sens.(threshold 0.9)	MC-Fold-DP	1.166E-13
MC-Fold-DP	CycleFold_thresholded	PPV(threshold 0.95)	none	nan
MC-Fold-DP	CycleFold_thresholded	sens.(threshold 0.95)	MC-Fold-DP	3.580E-15
MC-Fold-DP	CycleFold_thresholded	PPV(threshold 0.99)	none	nan
MC-Fold-DP	CycleFold_thresholded	sens.(threshold 0.99)	MC-Fold-DP	3.732E-17
CycleFold	CycleFold_thresholded	PPV(threshold 0.5)	none	4.042E-01
CycleFold	CycleFold_thresholded	sens.(threshold 0.5)	CycleFold	3.014E-03
CycleFold	CycleFold_thresholded	PPV(threshold 0.6)	none	4.589E-01
CycleFold	CycleFold_thresholded	sens.(threshold 0.6)	CycleFold	2.800E-04
CycleFold	CycleFold_thresholded	PPV(threshold 0.7)	none	2.724E-01
CycleFold	CycleFold_thresholded	sens.(threshold 0.7)	CycleFold	8.255E-06
CycleFold	CycleFold_thresholded	PPV(threshold 0.8)	none	6.215E-01
CycleFold	CycleFold_thresholded	sens.(threshold 0.8)	CycleFold	5.892E-07
CycleFold	CycleFold_thresholded	PPV(threshold 0.85)	none	6.237E-01
CycleFold	CycleFold_thresholded	sens.(threshold 0.85)	CycleFold	2.056E-08
CycleFold	CycleFold_thresholded	PPV(threshold 0.9)	none	9.164E-01
CycleFold	CycleFold_thresholded	sens.(threshold 0.9)	CycleFold	4.936E-10
CycleFold	CycleFold_thresholded	PPV(threshold 0.95)	none	nan
CycleFold	CycleFold_thresholded	sens.(threshold 0.95)	CycleFold	4.589E-11
CycleFold	CycleFold_thresholded	PPV(threshold 0.99)	none	nan
CycleFold	CycleFold_thresholded	sens.(threshold 0.99)	CycleFold	2.510E-12
CycleFold_constrained	CycleFold_thresholded	PPV(threshold 0.5)	none	4.801E-01
CycleFold_constrained	CycleFold_thresholded	sens.(threshold 0.5)	CycleFold_constrained	4.737E-05

CycleFold_constrained	CycleFold_thresholded	PPV(threshold 0.6)	none	5.583E-01
CycleFold_constrained	CycleFold_thresholded	sens.(threshold 0.6)	CycleFold_constrained	4.071E-06
CycleFold_constrained	CycleFold_thresholded	PPV(threshold 0.7)	none	1.214E-01
CycleFold_constrained	CycleFold_thresholded	sens.(threshold 0.7)	CycleFold_constrained	9.653E-08
CycleFold_constrained	CycleFold_thresholded	PPV(threshold 0.8)	none	5.770E-01
CycleFold_constrained	CycleFold_thresholded	sens.(threshold 0.8)	CycleFold_constrained	5.678E-09
CycleFold_constrained	CycleFold_thresholded	PPV(threshold 0.85)	none	5.795E-01
CycleFold_constrained	CycleFold_thresholded	sens.(threshold 0.85)	CycleFold_constrained	8.501E-11
CycleFold_constrained	CycleFold_thresholded	PPV(threshold 0.9)	none	1.000E+00
CycleFold_constrained	CycleFold_thresholded	sens.(threshold 0.9)	CycleFold_constrained	5.610E-13
CycleFold_constrained	CycleFold_thresholded	PPV(threshold 0.95)	none	nan
CycleFold_constrained	CycleFold_thresholded	sens.(threshold 0.95)	CycleFold_constrained	2.010E-14
CycleFold_constrained	CycleFold_thresholded	PPV(threshold 0.99)	none	nan
CycleFold_constrained	CycleFold_thresholded	sens.(threshold 0.99)	CycleFold_constrained	2.578E-16
