# Supplementary material for: Base pair probability estimates improve the prediction accuracy of RNA non-canonical base pairs
Source: PLoS Comput Biol. 2017 Nov 6;13(11):e1005827. doi: 10.1371/journal.pcbi.1005827 (PMC5690697; doi:10.1371/journal.pcbi.1005827)
Supplement: S7 Table — (PDF) [file pcbi.1005827.s008.pdf]

Supporting Table S7: Statistical comparison of CycleFold (MFE) partition function with canonical constraints against energy minimization algorithms for non-canonical pairs. If $p < 0.05$, the name of the program with significantly higher performance is provided.

program 1	program 2	metric	Significantly better performer	p value
MC-Fold	CycleFold_thresholded	PPV(threshold 0.5)	CycleFold_thresholded	2.730E-04
MC-Fold	CycleFold_thresholded	sens.(threshold 0.5)	CycleFold_thresholded	2.413E-03
MC-Fold	CycleFold_thresholded	PPV(threshold 0.6)	CycleFold_thresholded	1.141E-04
MC-Fold	CycleFold_thresholded	sens.(threshold 0.6)	CycleFold_thresholded	4.984E-02
MC-Fold	CycleFold_thresholded	PPV(threshold 0.7)	CycleFold_thresholded	8.773E-05
MC-Fold	CycleFold_thresholded	sens.(threshold 0.7)	none	7.256E-01
MC-Fold	CycleFold_thresholded	PPV(threshold 0.8)	CycleFold_thresholded	3.546E-03
MC-Fold	CycleFold_thresholded	sens.(threshold 0.8)	MC-Fold	1.532E-03
MC-Fold	CycleFold_thresholded	PPV(threshold 0.85)	CycleFold_thresholded	1.620E-03
MC-Fold	CycleFold_thresholded	sens.(threshold 0.85)	MC-Fold	1.342E-04
MC-Fold	CycleFold_thresholded	PPV(threshold 0.9)	CycleFold_thresholded	2.658E-02
MC-Fold	CycleFold_thresholded	sens.(threshold 0.9)	MC-Fold	7.534E-08
MC-Fold	CycleFold_thresholded	PPV(threshold 0.95)	CycleFold_thresholded	4.571E-03
MC-Fold	CycleFold_thresholded	sens.(threshold 0.95)	MC-Fold	2.642E-09
MC-Fold	CycleFold_thresholded	PPV(threshold 0.99)	none	1.828E-01
MC-Fold	CycleFold_thresholded	sens.(threshold 0.99)	MC-Fold	2.504E-19
MC-Fold-DP	CycleFold_thresholded	PPV(threshold 0.5)	CycleFold_thresholded	4.329E-03
MC-Fold-DP	CycleFold_thresholded	sens.(threshold 0.5)	none	9.300E-01
MC-Fold-DP	CycleFold_thresholded	PPV(threshold 0.6)	CycleFold_thresholded	7.320E-03
MC-Fold-DP	CycleFold_thresholded	sens.(threshold 0.6)	none	6.778E-01
MC-Fold-DP	CycleFold_thresholded	PPV(threshold 0.7)	CycleFold_thresholded	9.266E-03
MC-Fold-DP	CycleFold_thresholded	sens.(threshold 0.7)	none	1.932E-01
MC-Fold-DP	CycleFold_thresholded	PPV(threshold 0.8)	none	9.304E-02

MC-Fold-DP	CycleFold_thresholded	sens.(threshold 0.8)	MC-Fold-DP	8.381E-04
MC-Fold-DP	CycleFold_thresholded	PPV(threshold 0.85)	none	8.203E-02
MC-Fold-DP	CycleFold_thresholded	sens.(threshold 0.85)	MC-Fold-DP	1.611E-04
MC-Fold-DP	CycleFold_thresholded	PPV(threshold 0.9)	none	1.524E-01
MC-Fold-DP	CycleFold_thresholded	sens.(threshold 0.9)	MC-Fold-DP	4.539E-07
MC-Fold-DP	CycleFold_thresholded	PPV(threshold 0.95)	CycleFold_thresholded	1.506E-02
MC-Fold-DP	CycleFold_thresholded	sens.(threshold 0.95)	MC-Fold-DP	3.104E-08
MC-Fold-DP	CycleFold_thresholded	PPV(threshold 0.99)	none	4.226E-01
MC-Fold-DP	CycleFold_thresholded	sens.(threshold 0.99)	MC-Fold-DP	4.765E-16
CycleFold	CycleFold_thresholded	PPV(threshold 0.5)	CycleFold_thresholded	1.391E-04
CycleFold	CycleFold_thresholded	sens.(threshold 0.5)	CycleFold_thresholded	2.066E-03
CycleFold	CycleFold_thresholded	PPV(threshold 0.6)	CycleFold_thresholded	1.634E-04
CycleFold	CycleFold_thresholded	sens.(threshold 0.6)	none	1.244E-01
CycleFold	CycleFold_thresholded	PPV(threshold 0.7)	CycleFold_thresholded	9.761E-05
CycleFold	CycleFold_thresholded	sens.(threshold 0.7)	none	8.161E-01
CycleFold	CycleFold_thresholded	PPV(threshold 0.8)	CycleFold_thresholded	6.922E-03
CycleFold	CycleFold_thresholded	sens.(threshold 0.8)	CycleFold	1.405E-04
CycleFold	CycleFold_thresholded	PPV(threshold 0.85)	CycleFold_thresholded	5.457E-03
CycleFold	CycleFold_thresholded	sens.(threshold 0.85)	CycleFold	5.136E-06
CycleFold	CycleFold_thresholded	PPV(threshold 0.9)	none	8.493E-02
CycleFold	CycleFold_thresholded	sens.(threshold 0.9)	CycleFold	5.862E-10
CycleFold	CycleFold_thresholded	PPV(threshold 0.95)	CycleFold_thresholded	1.884E-02
CycleFold	CycleFold_thresholded	sens.(threshold 0.95)	CycleFold	1.096E-11
CycleFold	CycleFold_thresholded	PPV(threshold 0.99)	none	1.818E-01
CycleFold	CycleFold_thresholded	sens.(threshold 0.99)	CycleFold	4.588E-20
