# Supplementary material for: Base pair probability estimates improve the prediction accuracy of RNA non-canonical base pairs
Source: PLoS Comput Biol. 2017 Nov 6;13(11):e1005827. doi: 10.1371/journal.pcbi.1005827 (PMC5690697; doi:10.1371/journal.pcbi.1005827)
Supplement: S8 Table — (PDF) [file pcbi.1005827.s009.pdf]

Supporting Table S8: Statistical comparison of performance between CycleFold-TurboFold and plmc. If p < 0.05, the name of the program with significantly higher performance is provided.

program 1	Program 2	metric	Significantly better performer	P value
CycleFold	plmc	PPV(canonical)	CycleFold	0.00278
CycleFold	plmc	Sens. (canonical)	none	0.360
CycleFold	plmc	PPV (non-canonical)	none	0.0998
CycleFold	plmc	Sens. (non-canonical)	none	0.149
