# Supplementary material for: Base pair probability estimates improve the prediction accuracy of RNA non-canonical base pairs
Source: PLoS Comput Biol. 2017 Nov 6;13(11):e1005827. doi: 10.1371/journal.pcbi.1005827 (PMC5690697; doi:10.1371/journal.pcbi.1005827)
Supplement: S9 Table — (PDF) [file pcbi.1005827.s010.pdf]

Supporting Table S9. Time Benchmarks. CycleFold is compared to the standard (canonical pair) partition function in RNAstructure 6.0 (called partition) or to the standard (canonical pair) TurboFold in RNAstructure 6.0. The packages were built with GCC 4.9.2 in Debian Linux 8.8. The processor is a 4 core Intel Core i7-3770S CPU (3.10GHz) and the machine has 32 GB of RAM. Benchmarks were performed using serial (one compute core) versions of the code.

Sequence:	Mode:	Length: (nts)	CycleFold Time: (s)	Canonical Time: (s)
8psh	Single Sequence	16	0.5	0.1
429d	Single Sequence	48	2.0	0.1
2gdi	Single Sequence	160	24.5	0.5
2v3c	Single Sequence	192	36.4	0.7
MVE virus nuclease resistant RNA	TurboFold – 5 sequences	71.4 mean	77.2	2.4
MVE virus nuclease resistant RNA	TurboFold – 10 sequences	69.2 mean	176.6	6.4
SRP hairpin domain	TurboFold – 5 sequences	143.8 mean	679.2	31.8
SRP hairpin domain	TurboFold – 10 sequences	140.6 mean	2120.9	84.8
Twister ribozyme	TurboFold – 5 sequences	65.4 mean	61.4	2.1
Twister ribozyme	TurboFold – 10 sequences	68.1 mean	168.2	6.2
